# Earliest Pottery on New Guinea Mainland Reveals Austronesian Influences in Highland Environments 3000 Years Ago

**DOI:** 10.1371/journal.pone.0134497

**Published:** 2015-09-02

**Authors:** Dylan Gaffney, Glenn R. Summerhayes, Anne Ford, James M. Scott, Tim Denham, Judith Field, William R. Dickinson

**Affiliations:** 1 Department of Anthropology and Archaeology, University of Otago, Dunedin, 9054, New Zealand; 2 Department of Geology, University of Otago, Dunedin, 9054, New Zealand; 3 School of Archaeology and Anthropology, College of Arts and Social Sciences, Australian National University, Canberra, ACT, 0200, Australia; 4 School of Biological, Earth and Environmental Sciences, The University of New South Wales, Kensington, NSW, 2052, Australia; 5 Department of Geoscience, University of Arizona, Tucson, AZ, 85721, United States of America; University of Otago, NEW ZEALAND

## Abstract

Austronesian speaking peoples left Southeast Asia and entered the Western Pacific c.4000-3000 years ago, continuing on to colonise Remote Oceania for the first time, where they became the ancestral populations of Polynesians. Understanding the impact of these peoples on the mainland of New Guinea before they entered Remote Oceania has eluded archaeologists. New research from the archaeological site of Wañelek in the New Guinea Highlands has broken this silence. Petrographic and geochemical data from pottery and new radiocarbon dating demonstrates that Austronesian influences penetrated into the highland interior by 3000 years ago. One potsherd was manufactured along the northeast coast of New Guinea, whereas others were manufactured from inland materials. These findings represent the oldest securely dated pottery from an archaeological context on the island of New Guinea. Additionally, the pottery comes from the interior, suggesting the movements of people and technological practices, as well as objects at this time. The antiquity of the Wañelek pottery is coincident with the expansion of Lapita pottery in the Western Pacific. Such occupation also occurs at the same time that changes have been identified in subsistence strategies in the archaeological record at Kuk Swamp suggesting a possible link between the two.

## Introduction

In the Western Pacific, the appearance of new forms of material culture and domesticated animals, coupled with changes to food production systems 3300–3000 years ago is often considered to mark the interaction between Austronesian speaking populations moving out of Southeast Asia into the islands of the Pacific, and indigenous non-Austronesian speakers already established in these areas [1]. These changes have been well documented in the Bismarck Archipelago and areas further east, where the archaeological signature of this interaction is known as the Lapita Cultural Complex. Lapita is characterised by a distinctively decorated pottery component. This pottery appears in the Bismarck Archipelago by 3300 years ago, an area that had been occupied since the late Pleistocene. Its arrival in Remote Oceania on the other hand marks a colonising population entering a new area for the first time and sees with it the introduction of a translocated economy of cultigens (banana, taro, yam, breadfruit, and sugarcane), largely from the New Guinea region, and domesticated animals (pig, dog, and chicken), ultimately of an Asian origin, along with a unique material culture, including obsidian, imported from sources in the Bismarck Archipelago. The current consensus is that, to arrive in the Western Pacific, Austronesian speaking peoples skirted the already populated coastal areas of New Guinea [2–3], settling on the islands offshore in the Bismarck Archipelago, with their appearance on mainland New Guinea occurring much later in time, 2800 years ago, on the south coast of Papua [4–6]. Pottery excavated in the highland interior of New Guinea, however, holds clues to assessing this early Austronesian influence on the mainland.

### Wañelek

**Fig 1:**
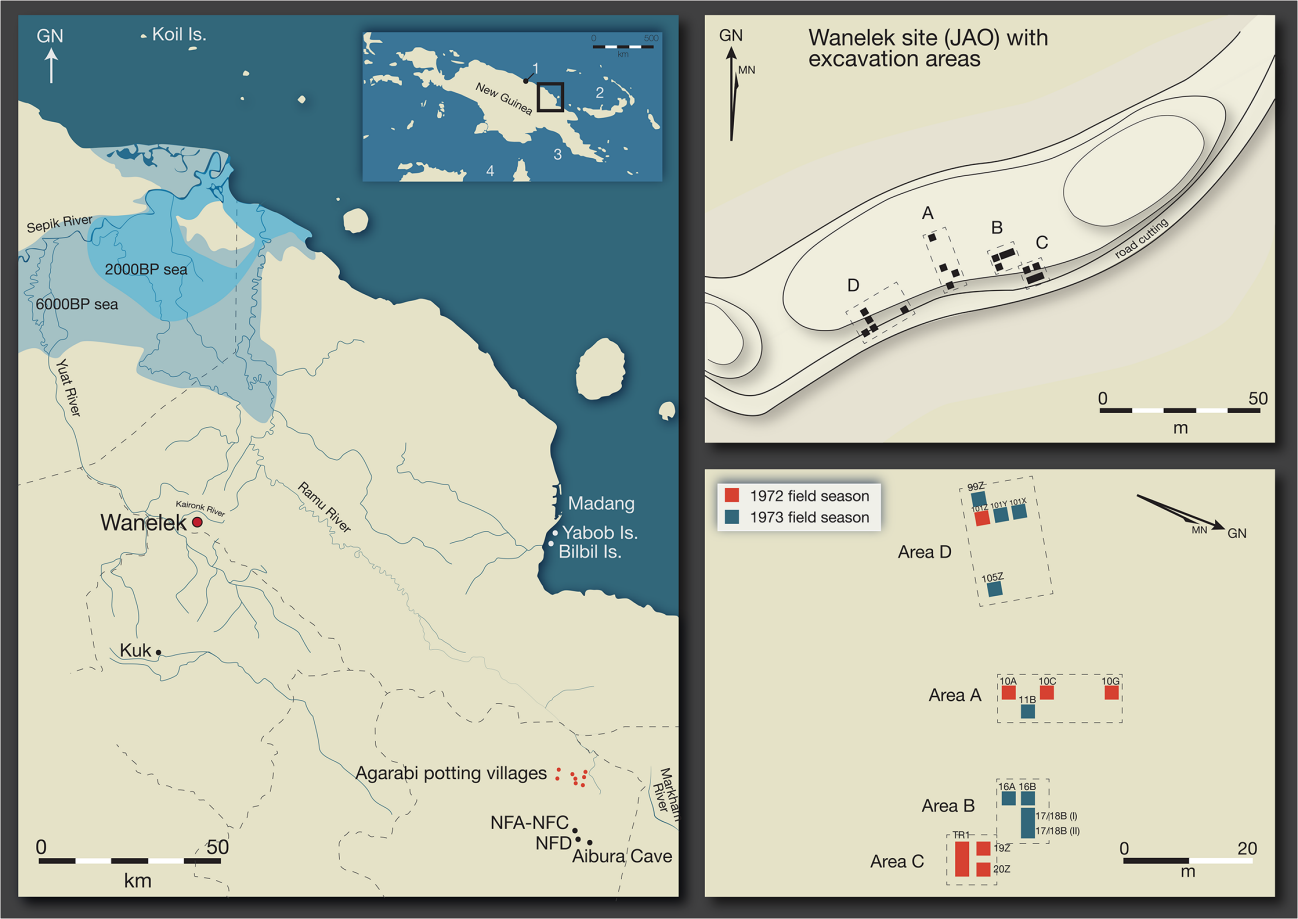
Locations mentioned in the text. Left: The northeast part of New Guinea showing Wañelek, Kuk, NFA, NFB, NFC, NFD, and Aibura Cave sites in the Highlands. Note the Sepik-Ramu Inland Sea at 6000BP and 2000BP, the contemporary highland Agarabi potting villages, Yabob and Bilbil Islands just off the Madang/northeast coast, and Koil Island off the north coast. Inset shows New Guinea with 1. Aitape, 2. the Bismarck Archipelago, 3. the south Papuan coast, and 4. Australia; Above right: Plan of Wañelek site showing excavation areas and ridge contours (adapted from Bulmer [7]); Below right: Wañelek excavation areas showing the 1972 and 1973 excavation units (adapted from Bulmer [7]).

Pottery at the Highlands site of Wañelek (site code: JAO) comes from 3,000 year old contexts. Located on an open ridge top in the Kaironk Valley of the Bismarck Schrader Ranges, Wañelek is some 1710m above sea level (Fig 1) [7]. Its location is important as it is sited on the fringes of the Central Highlands valley systems, with the Ramu plains to the north forming a major ethnographically recorded trade corridor between higher and lower altitudes [8]. From 6000–2000 years ago the site would have been much closer to the coast as the Sepik-Ramu inland sea extended significantly inland [9]. In 1972 and 1973 Susan Bulmer excavated four areas of the site and initial radiocarbon results suggested that at least four separate occupations spanned from the Last Glacial Maximum though to the late Holocene [10]. Although there is no direct evidence for agriculture, the occupants during the late Holocene employed a technology of pottery, polished axes, and stemmed slate tools, distinct from earlier technologies used at the site [11–15]. The finds at Wañelek are highly significant as they allow us to assess one possible route for the transmission of agricultural crops and associated technologies into the Highlands. This route has been hypothesised [16] but never demonstrated.

Redating of charcoal samples from two areas of the site (Areas B and D) was used to investigate the antiquity of the Wañelek pottery. The integration of new and old dates for Wañelek indicate good chronostratigraphic integrity for each of the four excavation areas, namely radiocarbon dates become older with depth (Table 1). A major focus has been to date charcoal from the fills of archaeological features because they provide a more robust basis for interpretation as opposed to charcoal dispersed within major stratigraphic units. Of greatest significance for understanding the antiquity of the ceramic assemblage at Wañelek is the consistent date for a primary occupation around c. 3000 cal BP, which is evident in multiple dated feature fills and associated contexts from Areas B and C.

**Table 1:** Distribution of mid-late Holocene radiocarbon dates and pottery sherds at Wañelek by excavation area and unit. This demonstrates the secure chronostratigraphical context for the Wañelek pottery sherds during the main c.3000 year old occupation phase.

Area	Excavation unit	Unit layer	Laboratory code*	Material	δC13^†^	Conventional radiocarbon age BP	Calibrated (1σ) date range BP^‡^	Calibrated (2σ) date range BP^‡^	Pottery present
A	11B	9	GX-3333B	Charcoal	-	3840±175	3930–4437	3721–4807	Layer 8
	10A	10	I-6860	Charcoal	-	5455±105	6020–6313	5944–6409	-
B	16B	6 (fireplace)	GX-3330	Charcoal	-	3225±180	3171–3681	2950–3863	Layer 6
	17-18B(I)	5	GX-3327B	Charcoal	-	3230±230	3081–3700	2810–3984	-
	17-18B(I)	7 (oven pit)^§^	GX-3326	Charcoal	-	3430±175	3450–3870	3260–4148	-
	17-18B(I)	8	Wk-20407	Charcoal	-25.5±0.2	2981±34	3079–3236	3009–3323	-
	17-18B(I)	11 (posthole)	Wk-20409	Charcoal	-25.9±0.2	4239±38	4713–4854	4629–4867	-
	17-18B(II)	5	Wk-20410	Charcoal	-27.8±0.2	3052±34	3222–3336	3164–3361	Layer 2
	17-18B(II)	8 (pit top of layer) ^§^	GX-3332	Charcoal	-	3170±210	3075–3613	2799–3844	Layer 2
C	Trench 1	4 (fireplace)	I-6861	Charcoal	-	2840±90	2795–3061	2757–3205	Layer 5 (top of pit fill)
	Trench 1	5 (deepest fill of pit)	I-6859	Charcoal	-	2865±90	2846–3136	2766–3212	Layer 5 (top of pit fill)
D	105Z	1	Wk-22058	Charcoal	-15.5±0.2	135.1±0.5	22–265	20–267	Layer 5–7
	105Z	5	Wk-22060	Charcoal	-25.2±0.2	3697±30	3985–4084	3929–4146	Layer 5–7
	101Y	3	Wk-20401	Charcoal	-27.3±0.2	872±36	732–896	698–907	Layer 2–3

*I-6861, I-6859, I-6860 dated at Teledyne Isotopes, 1973. ‘GX’ samples dated (and redated at least once) from 1973–1974 at Krueger Enterprises, Inc. Geochron Laboratories Division, Cambridge, MA. ‘Wk’ samples dated at Waikato Radiocarbon Dating Laboratory, New Zealand in 2007 for the current paper.

^†^ δC13 correction not originally made. Here an estimate of -25.0±2 is applied to Bulmer’s conventional C14 age for charcoal before calibration [17].

^‡^ Calibrated dates generated by *Calib 6*.*1*.*0*. [18, 19].

^§^ Layer contexts of GX-3332 and GX-3326 recently revised by S. Bulmer (unpublished personal correspondence).

**Fig 2:**
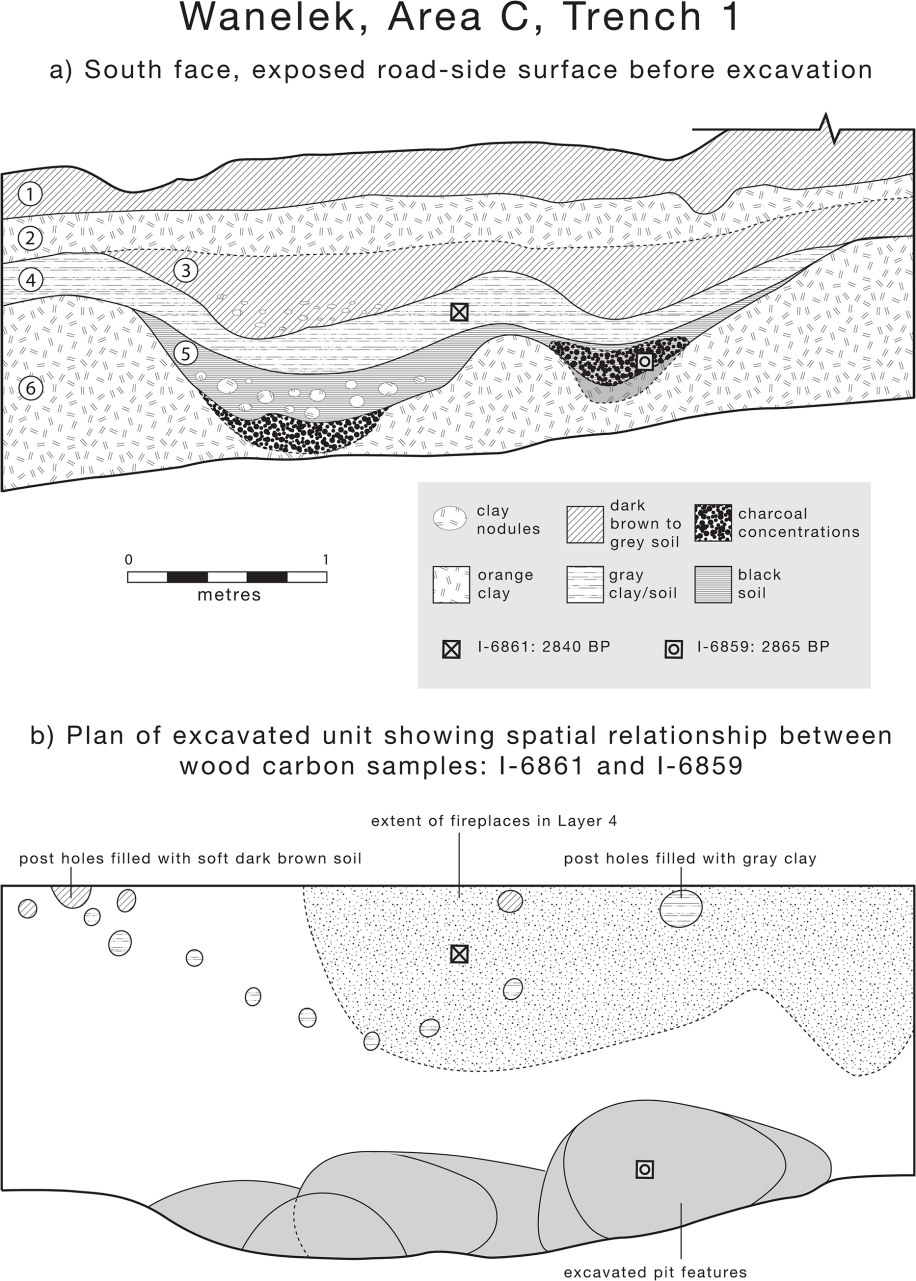
Wañelek, Area C, Trench 1, showing a) the south face of the unit exposed by a road cutting, before excavation, and b) a plan of the trench indicating the location of pit features. Note the locations of carbon samples I-6859 and I-6861 giving secure provenance for sherd W52 (adapted from Bulmer [7]).

**Fig 3:**
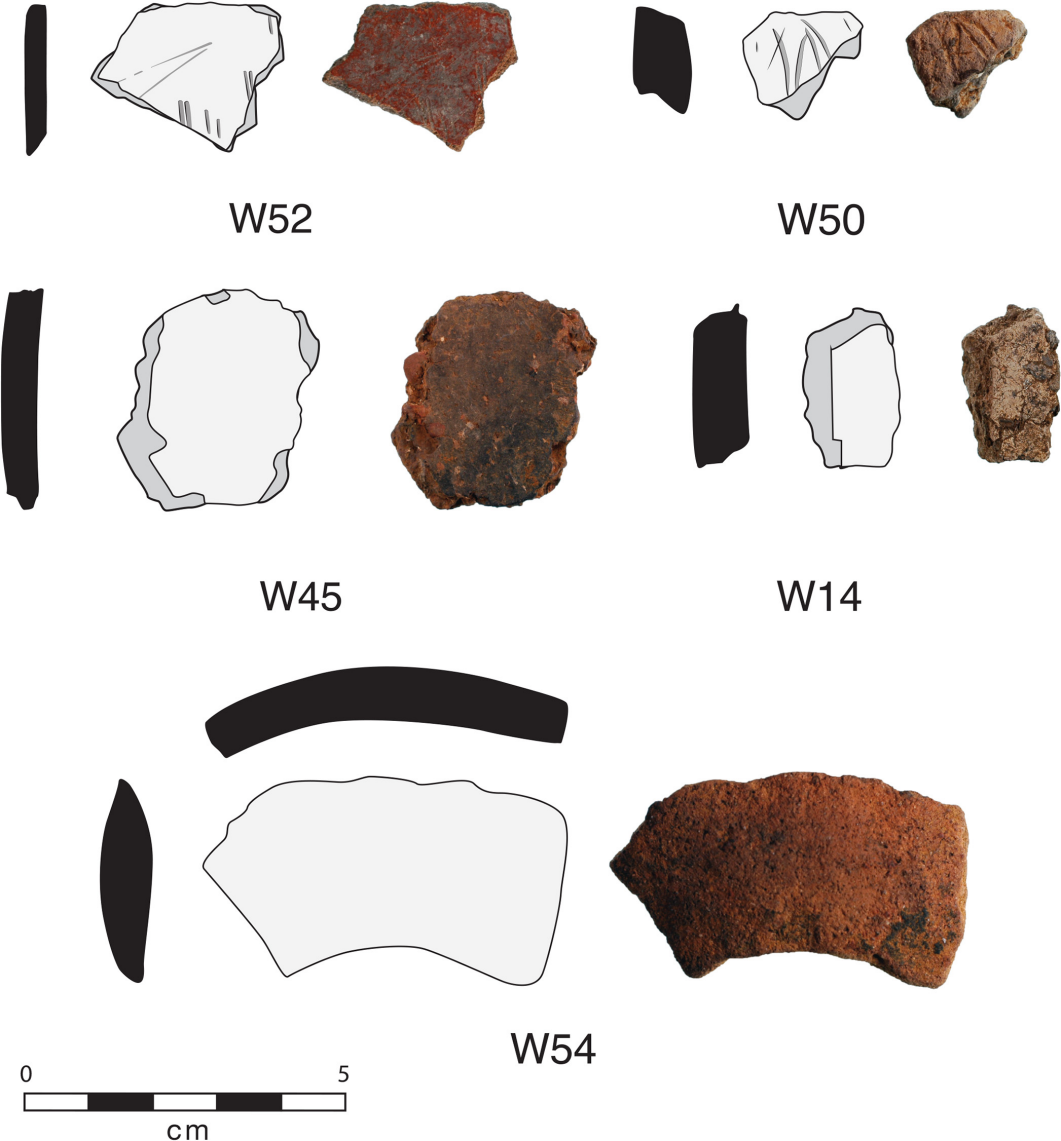
Examples of Wañelek pottery. W52: paddle and anvil made body sherd with red slip and incised decoraton; W50: body sherd with fingernail incision; W45: plain body sherd with red burnish or slip; W14: plain body sherd; W54: possible broken coil or weathered rim sherd.

Table 2 describes each potsherd with its associated provenance. Of most significance is a sherd of red-slipped pottery with incised decoration (W52), in a pit in Area C, Trench 1, associated with this occupation (Figs 2 and 3). The lower fills of the pit (I-6859) and an overlying buried ground surface (I-6861) are securely dated to ensure a robust chronological basis for interpretation. Additionally, one sherd (W45) was collected from a stratigraphic context associated with the main occupation in Area B, Unit 16B, around 3000 years old (GX-3330). Sherds W11-14 are from a stratigraphic layer associated with a slightly earlier occupation post-dating c.4000BP (Wk-22060). In Area D, other sherds, W50 and W55, are only c.700-910 cal BP (Wk-20401). Other sherds at the site are derived from less securely dated contexts: W16 and W54 post-date c. 3720–4810 cal BP (GX-3333B) in Area A; W6 and W7 post-date the main occupation in Area B; and W10 post-dates c.700-910 cal BP (Wk 20401), whereas W9 and W35 are likely associated with an earlier occupation post-dating c. 4000 cal BP in Area D given they were both excavated from secure stratigraphic layers either adjacent to or deeper than Wk-22060.

**Table 2:** Wañelek pottery artefacts and associated stratigraphic provenance. Estimates for dates of each potsherd are provided by assessing new radiocarbon dates by area.

Potsherd #	Area	Excavation Unit	Excavation Layer	Probable age
W1	Unstratified			?
W2	Unstratified			?
W3	Unstratified			?
W4	Unstratified			?
W5	Unstratified			?
W6	B	17/18B(II)	Layer 2	< 3052BP
W7	B	17/18B(II)	Layer 2	<3052BP
W9	D	101Z	Layer 5	c.3697BP
W10	D	101Y	Layer 2	<872BP
W11	D	105Z	Layer 5	c.3697BP
W12	D	105Z	Layer 5	c.3697BP
W13	D	105Z	Layer 5	c.3697BP
W14	D	105Z	Layer 5	c.3697BP
W16	A	11B	Layer 8	<3840BP
W35	D	105Z	Layer 7	>3697BP
W45	B	16B	Layer 6	c.3225BP
W50	D	101Y	Layer 3	c.872BP
W52	C	TR1	Layer 5 (pit feature)	c.2865BP, >2840BP
W54	A	10C	Layer 5	<3840BP
W55	D	101Y	Layer 3	c.872BP

As pottery was introduced into the Western Pacific by Austronesian speaking peoples and is all but invisible along the north coast of New Guinea until much later in time, the presence of pottery in 3,000 year old contexts in the remote highland interior of New Guinea provides a unique glimpse into the nature of the Austronesian expansion. The peoples moving into Remote Oceania carried with them an agricultural base derived from mainland New Guinea, not Southeast Asia. Most of their plant foods were domesticated in mainland New Guinea, suggestive of cultural integration in Roger Green’s Triple I model: Intrusion, Innovation, and Integration [20]. Therefore, the finds at Wañelek provide for the first time material evidence to address the interaction between Austronesian speakers and established indigenous populations from the north coast of New Guinea and adjacent highland areas.

A priority in understanding the nature of this interaction lies in identifying how the pottery was introduced. In order to identify the place of manufacture for these 3000 year old ceramics a series of analyses were conducted on the Wañelek pottery using petrographic analysis and Scanning Electron Microscope (SEM). Petrographic analysis of pottery sherds has been a proven method for interpreting prehistoric trade and interaction for over half a century [21], and along with geochemical charaterisation has been particularly successful in sourcing ceramics to their manufacturing areas in the Pacific [22–23]. This article presents the results of these analyses and discusses the implications for the archaeology of the Austronesian expansion.

## Materials and Methods

Based on a review of the Wañelek assemblage, a total of twenty pottery sherds were identified (contra recent reports by Bulmer [15] in which some weathered sandstones had been misidentified as pottery; see S1 Appendix for descriptions of the Wañelek pottery sherds). The sherds were small and very fragmented (average maximum dimension = 33mm). These were divided into macroscopic fabric categories, based on clay colour and temper type, assisted by 40x magnification. Twelve of these sherds were sampled for petrographic and geochemical analysis, selected in order to represent the diversity of fabric groups at Wañelek, as well as all stratigraphic levels that contained pottery.

Standard petrographic analysis was undertaken on the twelve sherds, which allowed for a general appraisal of the mineral inclusions. An additional quantitative chemical analysis of non-plastic minerals and the clay matrix was completed on a Zeiss Sigma field emission gun scanning electron microscope (FEG SEM) in the Otago Centre for Electron Microscopy (OCEM), using an XMax20 silicon drift energy dispersive X-ray (EDX) detector and AZtec acquisition and processing software. The FEG SEM produces high-resolution images and detects contrasts between areas with different elemental compositions and surface topographies. The EDX allows spot probe analysis and determines the elemental chemical composition of a point or zone on the sample [24].

Six areas of each sample were examined: three electron micrographs at 100x magnification to examine mineral inclusions; and three at 2000x to examine the clay matrix. Two different methods of analysis were used: 1) A spot point analysis of the clay matrix which collected elemental data at five points per view, and 2) map-scanning, which obtained x-ray data for an entire micrograph rather than particular points, was used to describe and quantify the non-plastic mineral inclusions.

The geochemical data of the clay matrix was organised using Principal Component Analysis (PCA). PCA is a multivariate statistical tool that groups samples by chemical similarities, transforming the original variables into uncorrelated principal components [25]. PCA data was standardised using MVARCH [26].

Permission to undertake this research was given by the National Museum and Art Gallery of Papua New Guinea (NMAG) in conjunction with the National Research Institute of Papua New Guinea. All archaeological material is temporarily held in the Department of Anthropology and Archaeology, University of Otago, after which it will be repatriated as agreed with the NMAG.

## Results

### Macroscopic fabric analysis

Seven macro-fabric groups are identifiable in the Wañelek assemblage (Table 3; S1 Appendix). This includes diverse clay types and non-plastic mineral inclusions, from small, well-sorted, artificially-added tempers, to large, poorly-sorted, natural lithic inclusions. Samples representative of Bulmer’s fabric groups 1–3 were identified, along with four other fabrics that have not previously been reported (fabric groups 4–7). From the assemblage, twelve sherds were selected for petrographic and chemical analysis.

**Table 3:** Macro-fabric groupings 1–7 identified in the Wañelek pottery assemblage.

Fabric group	No of sherds	Clay colour*	Major inclusions	Mean thickness	Decoration present	Surface finish
1	8	Pale brown (2.5Y 7/3)	Large quartz/feldspathic fragments and rounded lithic pebbles	8.43mm	Linear incision/fingernail impression	None
2	6	Strong brown (7.5YR 5/6)- Reddish yellow (7.5YR 6/6)	Small subangular light inclusions (quartz/feldspars); rounded black and red ferro-magnesiums	7.59mm	Plain	None
3	1	Brown (7.5YR 7/5)- reduced firing	Small angular-subangular ferro-magnesiums; minor light inclusions (quartz/feldspars)	3.72mm	Short linear incisions	Red slip
4	2	Dark yellowish brown (10YR 4/4)- light yellowish brown (10YR 6/4)	Small subangular light inclusions (quartz/feldspars) and small rounded ferro-magnesiums	7.14mm	Plain	None
5	2	Grayish brown (10YR 5/2)	Large rounded-sub angular red ferro-magnesium inclusions; small subangular light inclusions (feldspars)	4.30mm	Plain	Red slip
6	1	Reddish yellow (5YR 6/6)	Thin rounded lithic pebbles; small angular light inclusions (quartz)	5.85mm	Plain	Red slip
7	5	Very pale brown (10YR 8/4)	Quartz/feldspathic fragments and sub-angular lithic pebbles	8.09mm	Plain	None

*Munsell soil colour chart 2009

### Petrographic analysis

Fabric groups 1, 2, 4, 5, 6, and 7 contain poorly sorted, subangular sands that are probably natural inclusions in the clay sources, rather than being manually added tempers by potters. The inclusions are heterogeneous sands containing quartzo-feldspathic mineral grains and a variety of polycrystalline sedimentary and metasedimentary rock fragments that include quartzitic, fine-grained argillitic, and foliated metamorphic rocks (slate or phyllite).

Fabric group 3 (sherd W52) is mineralogically distinct from other samples and contains sand-sized grains of rock fragments and minerals set within a brown clay matrix. The rock fragments are dominantly of volcanic origin, commonly displaying trachyitic or porphyritic textures. Metamorphic rock fragments are absent. Single minerals present within the sherd are: amphibole, clinopyroxene, albitised plagioclase feldspar (with sericitic alteration, although twinning is still observable), quartz, orthopyroxene and Fe-oxides.

### SEM analysis of mineral inclusions

The geochemical identification of minerals (S1 Table; S2 Appendix) corresponds with the macro-fabric groupings and petrographic analysis, however minor inclusions (<1%) not described in the petrographic analysis are identifiable in some sherds. Feldspathic inclusions as single minerals and components of large rock fragments (some foliated) suggests that fabric groups 1, 2, 4, 5, 6, and 7 are mostly derived from metamorphic zones in the interior. Shell was not observed in any sample, therefore there is no direct link with manufacturing sites on the coast, however W52 (fabric group 3) is distinct in having well sorted inclusions of pyroxene and Fe-oxides, with fewer feldspars and large rock fragments.

### Chemical analysis of clays

**Fig 4:**
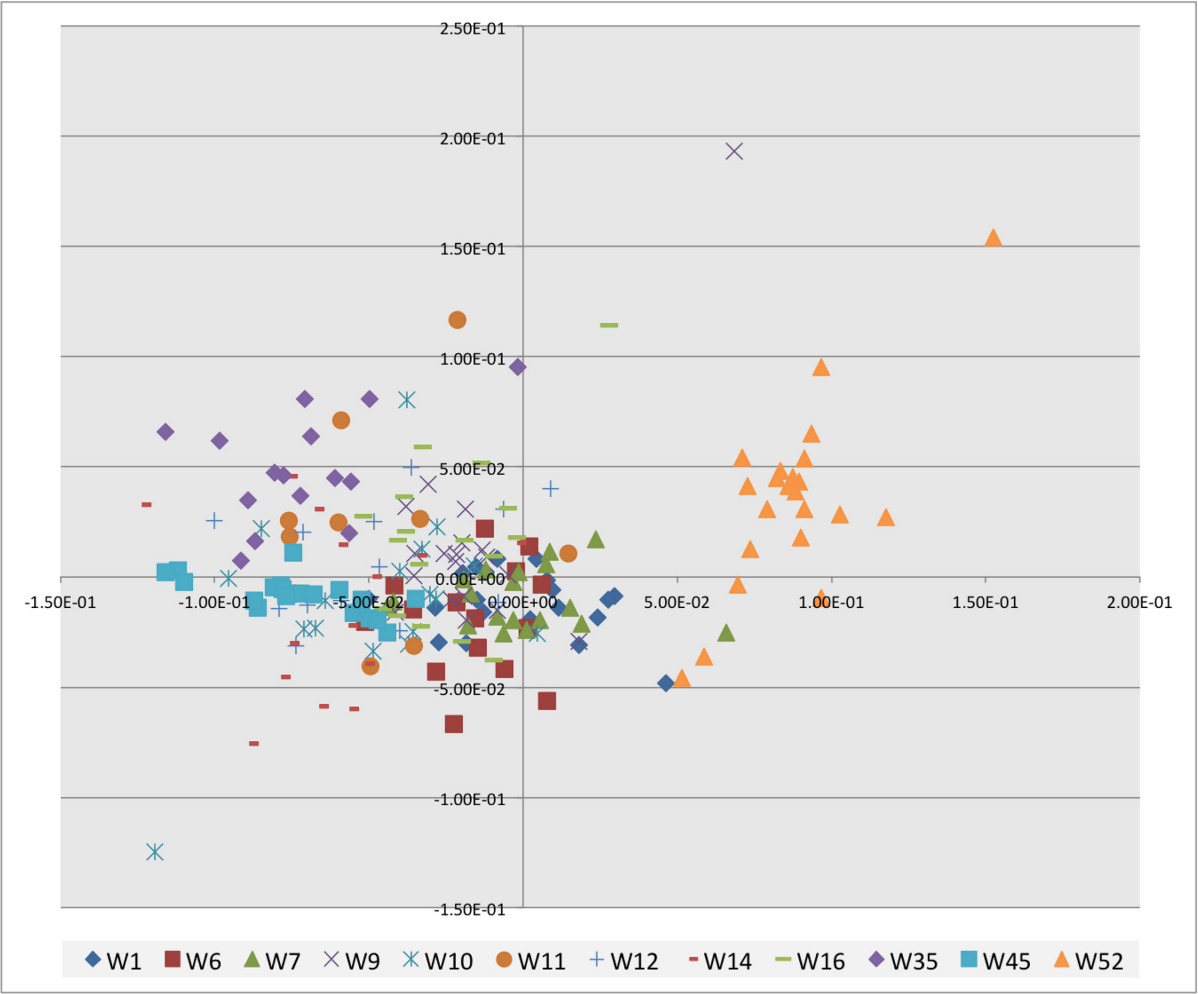
Plot of clay chemical PCA data in two dimensions showing the seperation of W52 away from other sherds.

The geochemical data of the clay matrix (S1 Dataset) was organised using Principal Component Analysis (PCA). Visual inspection of plotted data in two dimensions identifes a number of chemical groups (Fig 4). One sample, W52 (fabric group 3), separates out from the other sherds, which overlap with a considerable amount of intrasample variation. This suggests distinct clay provenance for W52 with similar chemical signatures for all other sherds.

## Discussion

The clay data suggest that fabric group 3 is distinct while all other fabrics derive from similar clay mineral sources. From the non-plastic mineralogical analyses of fabrics 1, 2, 4, 5, 6, and 7 there does not seem to be any specific signal of provenance for the temper sands, which may resemble sands from multiple areas within the New Guinea interior. Temper sourcing on continental landmasses presents a more severe challenge than temper sourcing on smaller islands, which virtually serve as point sources of derivative sediment. On larger landmasses, subregional rivers may tap multiple bedrock sources and mingle sediment of mixed geologic provenance along their courses.

The inland inclusions in the Wañelek sherds do not closely resemble inclusions in sherds from the Aitape coast of northern Papua New Guinea [27] nor in sherds from Koil Island offshore from Wewak on the north coast (Summerhayes pers. comms.). All those tempers associated with the coasts of Papua New Guinea contain distinctly greater proportions of volcaniclastic detritus derived from either the Torricelli mountain system parallel to the north coast or from the volcanic province of the Western Highlands, west and south of Mount Hagen. Many of the Wañelek mineral inclusions could be taken as generically representative of sands derived from the non-volcanic pre-Tertiary bedrock of the Papua New Guinea highland ranges. This indicates that fabrics 1, 2, 4, 5, 6, and 7 were either manufactured in the highland interior itelf or a significant distance inland from the north coast, around the foothill zones, with rivers draining from highland metamorphic zones.

Fabric 3 (W52) comfortably fits into the Andesitic arc temper group [22]. The absence of sedimentaries and metasedimentaries in W52 suggest it is not from the Sepik north coast, which is defined by heterogeneous lithic inclusions derived from the Torricelli Mountains. The inclusion of quartz derived from older parent rocks however suggests W52 was not produced on the small outlying islands of the Bismarck Archipelago, although this does not preclude the larger islands of New Britain and New Ireland. Mineralogical comparisons between W52 and ancestral Madang sherds from Yabob and Bilbil Islands suggest a very similar range of minerals with the exception that small amounts of orthopyroxene are present in W52 but not the ancestral Madang sherds. The most likely provenance for W52 is therefore somewhere on the northeast coast of New Guinea or nearby islands. The incised decoration and red slip along with paddle and anvil manufacture for W52 has parallels to both ancestral Madang and the Sepik traditions on the north coast [28–30]. Today, manually tempered and red-slipped wares are only made by Austronesian speaking communities in New Guinea (the one exception being Mailu on the south coast who produce manually tempered pots, but speak a non-Austronesian language), and paddle and anvil construction is almost exclusively practised by Austronesian speakers [31]. As potting is an extremely conservative practice [32–33] the occurrence of these three technologial factors- manual tempering, red slip, and paddle and anvil technique- is indicative of Austronesian manufacture.

Importantly, different fabric groups occur at different points in the chronological sequence (S2 Table). Fabric 1 is the most recent and appears to be associated with contexts of, or more recent than, c.700-910 cal. BP (Wk-20401). Fabric 3 can be confidiently dated to c.2800-3100 cal. BP (between I-6861 and I-6859). Fabric 5 is found in contexts relating to c.3000-3900 cal. BP (GX-3330). Fabrics 2, 4, and 7 seem to be the oldest sherds, associated with an occupation post-dating c.4000BP (Wk-22060). Fabric 6 has no associated date (surface collection).

### Comparative pottery sequences in the New Guinea Highlands

The only New Guinea Highlands locality where pottery making has been observed in the recent past is in the Agarabi language area, in Eastern Highlands province over 200km southeast of Wañelek [31, 34–35]. Agarabi pots were not widely traded, but share many stylistic similarities with inland coiled pottery, such as Usur or Rawa pots, which are ellipsoid with everted rims and pointed bases [8, 31]. The remainder of pottery observed ethnographically in the Central Highlands was imported from the hilly highland fringes, produced in villages near the Ramu River or on the coast [34–35]. Petrographic descriptions of modern pottery from highland and fringe highland zones, including the Agarabi area, suggests that pots were mostly made from local river clays with natural mineral and rock inclusions (S3 Table).

The Agarabi language is non-Austronesian, but their origin stories lie in the Markham Valley. Adzera communities who are Austronesian speakers, expanded up the Markham pushing out other Austronesian speakers such as the Mari. It is argued that refugees from these intrusions moved into the Arona Valley and over a number of centuries adopted the language of Gadsep speakers, a non-Austronesian language [36].

Currently, our knowledge of prehistoric pottery production in the Highlands, including the location of manufacture, is limited, with fewer than 400 excavated sherds (S4 Table), and firm chronologies limited to the last few centuries before European contact. Petrographic analysis has been completed for some of these sites. For example, NFA-NFD are four sites situated just north of the Lamari River valley in the Eastern Highlands and were occupied from 4000 years ago through to recent times. Mineralogical comparisons between sherds excavated at these sites and a modern Agarabi pot, indicate that most pottery used at these sites was of local Eastern Highlands manufacture. However, the earliest reliable date associated with pottery at these sites is only 185±80BP (I-7286) [37].

Four potsherds excavated at Aibura Cave, again in the Lamari River Valley, from a horizon dated to 770±110BP (GaK-622) (note Gakushuin radiocarbon lab results have been questioned for giving older than expected dates [38]), were also mineralogically similar to Agarabi pots. In this fabric, the clay was ‘lean’ and the inclusions of granite, quartzite, weathered feldspar (K-Na), quartz, and green hornblende, were poorly sorted, ranging in size from 0.1mm-1.5mm. The fabric of two other Aibura sherds were consistent with a modern sherd from Yabob, in which beach sand with an Upper Tertiary volcanic base was deliberately added for temper [39]. This demonstrated importation of pottery from the Madang coast to Aibura.

Pottery in the Eastern Highlands was produced locally, although some items originally manufactured on the coast were traded up into the higher altitudes. However, these potsherds date to within the last millennium and occur much later than the sherds excavated further west at Wañelek that first appear over 3000 years ago.

## Conclusions

The study presented here has shown for the first time that pottery was introduced to the New Guinea Highlands from the coastal region by the end of the fourth millennium BP. The pottery analysed from the Wañelek site in Kaironk River valley, shows a variety of sources. Of the twelve potsherds analysed, one was imported from the coast, while the remainder were produced within the interior from local resources. Whether the pottery, and the knowledge of its production, came with the original Austronesian speakers or was traded and copied will require further research from a wider area. What *is* known is that although the Sepik Inland Sea was ‘rapidly infilling’ by 3500 BP [9], when the first pottery arrived at Wañelek the shoreline was much closer to the site than it is today, allowing access up the Ramu and Yuat Rivers and across the divide. This demonstrates that early strategies of interaction were not solely focussed along the coast and offshore islands, but also up river valleys.

The pottery from Wañelek falls squarely within the period for the spread of Lapita within the Bismarck Archipelago and onwards to Remote Oceania [40–42]. It predates the appearance of Lapita pottery on the south Papuan coast. The occurrence of a sherd of red-slipped pottery with incised decoration from a securely dated 3000 year old context at Wañelek indicates that the highland interior of New Guinea was not isolated from broader socio-economic changes in the Western Pacific. Other sherds associated with this same 3000 year old occupation were made from inland materials. The Highlands were inter-connected with coastal regions and islands off the north coast, not only enabling the flow of pottery, but also enabling the movement of people and the technical ability to make it.

The social inter-connections between the Highlands and coast indicated at Wañelek provide a potential window onto other socio-economic changes. Agricultural practices in the Highlands were not occurring in isolation from the coast. The same trade networks that enabled the movement of pottery, as well as the know-how to make it, could have enabled the movement of cultivars as well. The transmissions of these new crops and associated technologies are apparent in changes to production at the Highlands site of Kuk [43]. As noted by Summerhayes [44], Bayliss-Smith [45] theorised that Austronesian influences were felt fairly quickly in the Highlands with a second production threshold reached at 2500 years ago that may be related to the arrival of Austronesian speakers on the coast. Certainly, gourds were introduced to New Guinea and adopted by horticulturalists in the Highlands by at least 3000–2000 years ago [46], and potentially earlier [47]. Conversely, other plants plausibly dispersed from the Highlands to lower altitudes and the circum-New Guinea region.

Such interactions sit well with the Intrusion, Innovation, and Integration model originally developed by Roger Green to account for the formation of the Lapita Cultutral Complex in the Bismarck Archipelago at roughly the same time period. On mainland New Guinea the identification of an Austronesian fingerprint by 3000 years ago in the Highlands, through the Sepik Inland Sea, breaks the silence of these past interactions and the invisibility of Austronesian integration.

## Supporting Information

S1 Appendix Illustration and photography of Wañelek pottery by fabric group. (PDF)

S2 Appendix SEM micrographs of Wañelek pottery by fabric group. (PDF)

S1 Dataset SEM chemical data for clay matrix of Wañelek pottery samples. (XLSX)

S1 Table Summary of macroscopic fabric groups and associated mineralogies by map scanning. Highlighted samples were sampled for SEM analysis.
(DOCX)

S2 Table Estimates for earliest dates of pottery fabric groups at Wañelek. (DOCX)

S3 Table Comparative fabric reports on modern pottery from the New Guinea Highlands and nearby areas. (DOCX)

S4 Table Archaeological pottery from excavated New Guinea Highland sites. (DOCX)
