# Supplementary material for: Earliest Pottery on New Guinea Mainland Reveals Austronesian Influences in Highland Environments 3000 Years Ago
Source: PLoS One. 2015 Sep 2;10(9):e0134497. doi: 10.1371/journal.pone.0134497 (PMC4557931; doi:10.1371/journal.pone.0134497)

S.I. Appendix I: Wañelek pottery by fabric group

Wanelek pottery- fabric group 1

W3
plain
body

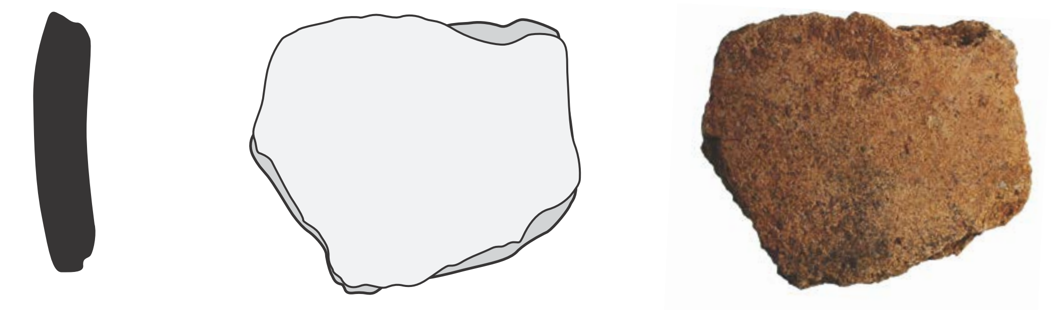

W10
plain
body

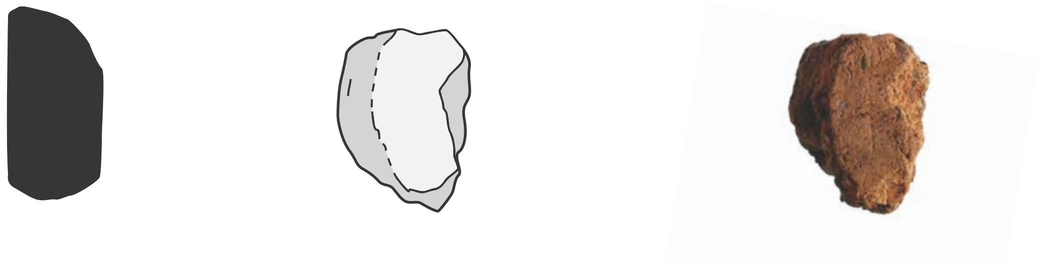

W16
plain
body

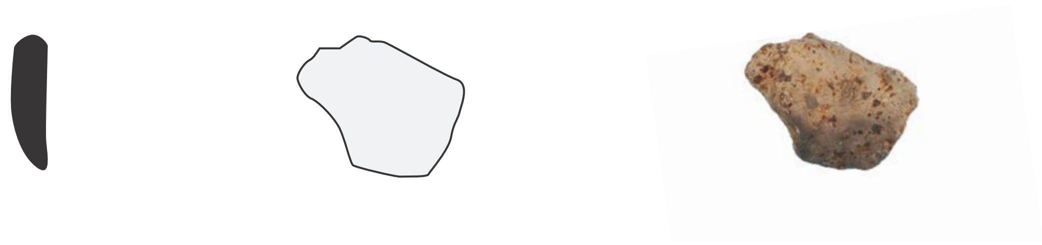

W50
decorated
body

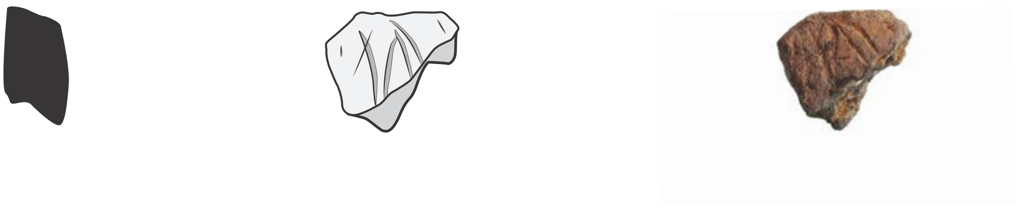

W55
decorated
rim

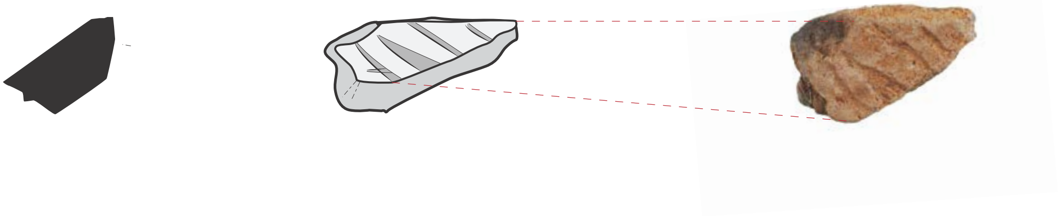

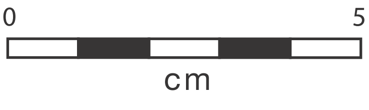

Wanelek pottery- fabric group 2

W2
plain
body

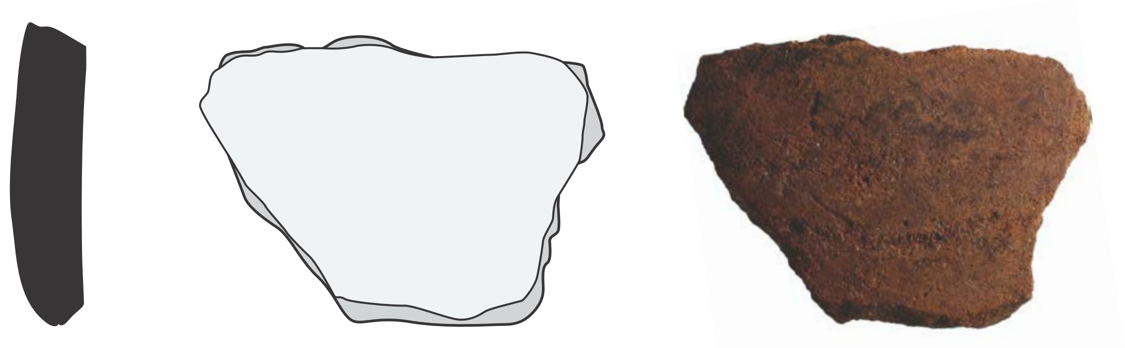

W4
plain
body

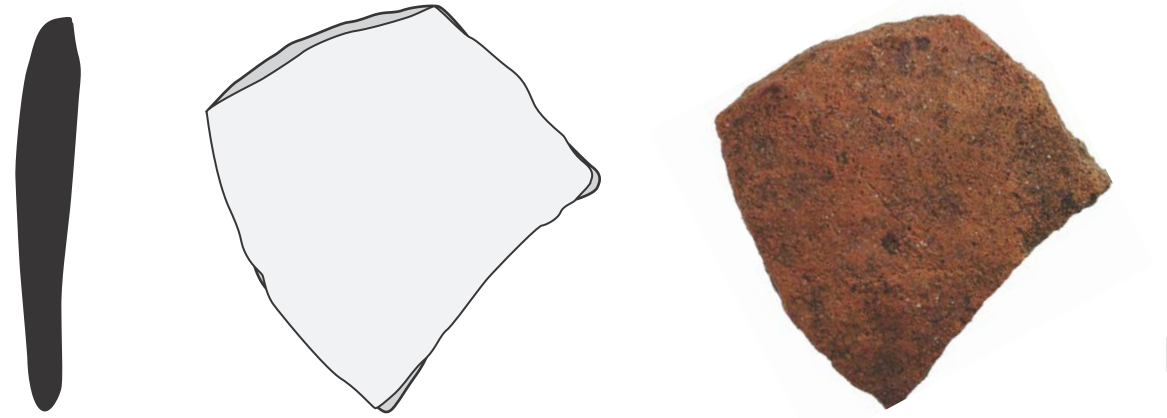

W5
plain
body

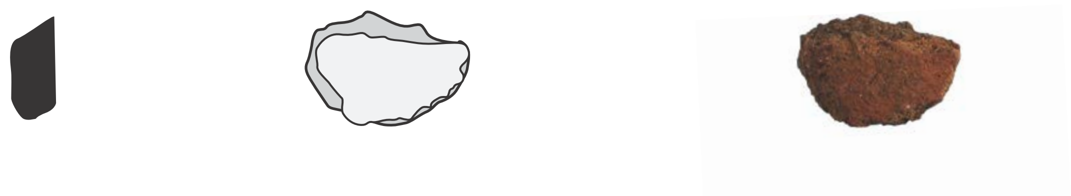

W13
plain
body

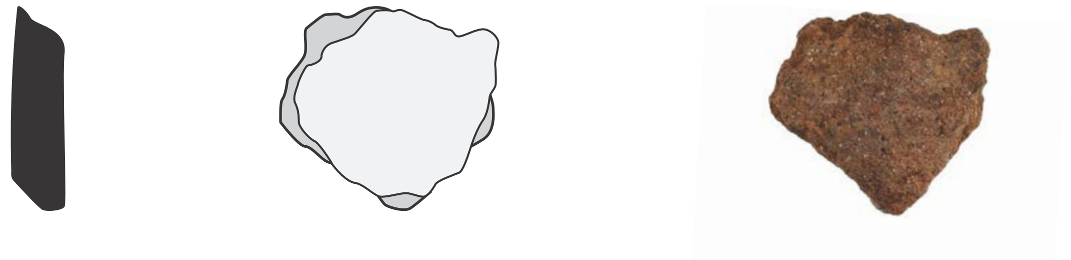

W35
plain
body

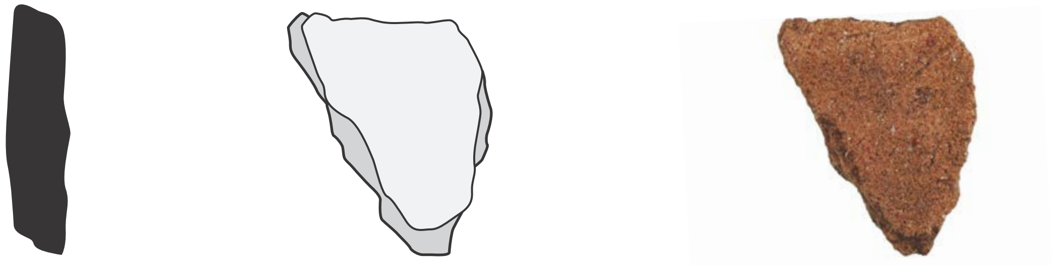

W54
broken
coil or
plain rim

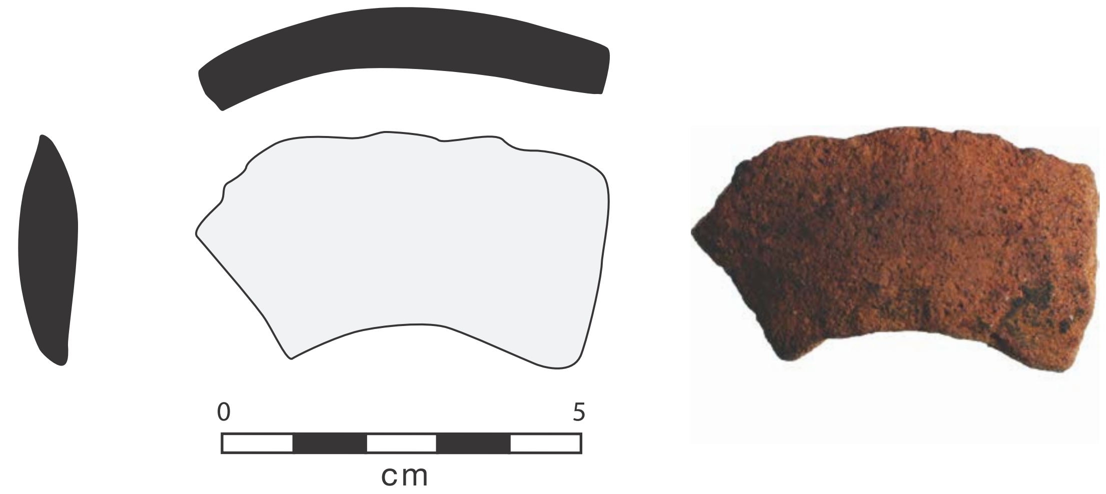

Wanelek pottery- fabric group 3

W52
decorated
body

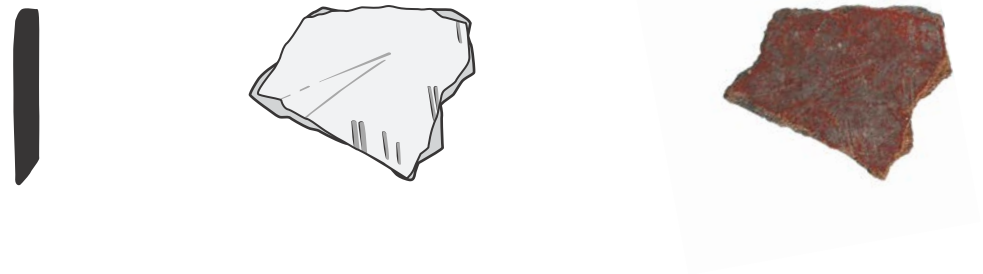

Wanelek pottery- fabric group 4

W6
plain
body

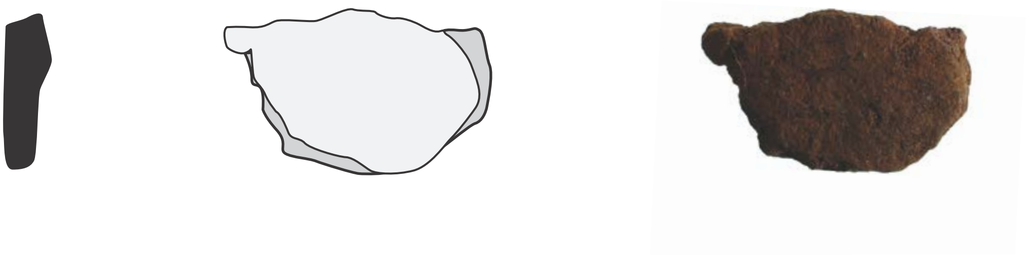

W9
plain
body

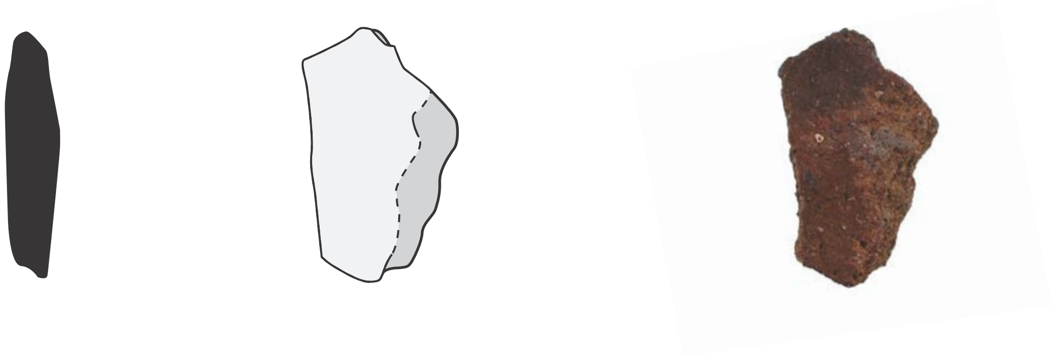

Wanelek pottery- fabric group 5

W7
plain
body

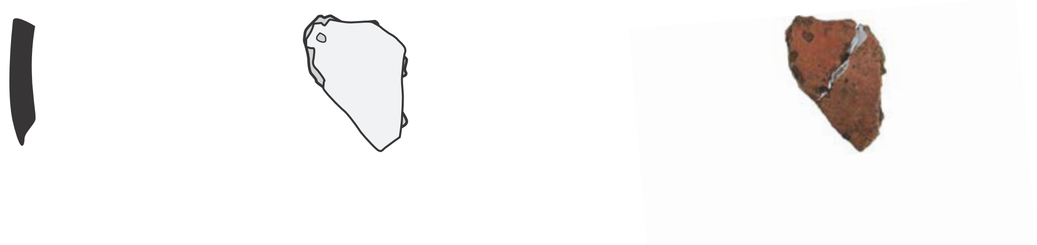

W45
plain
body

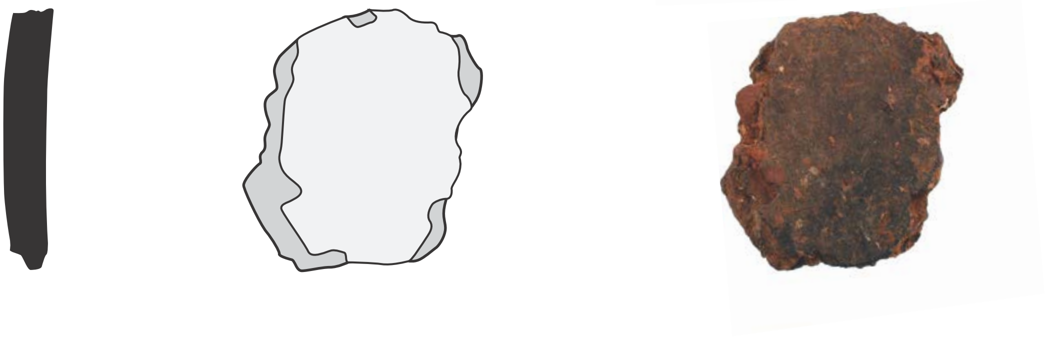

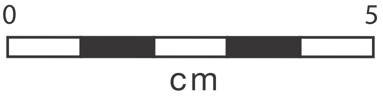

Wanelek pottery- fabric group 6

W1
plain
body

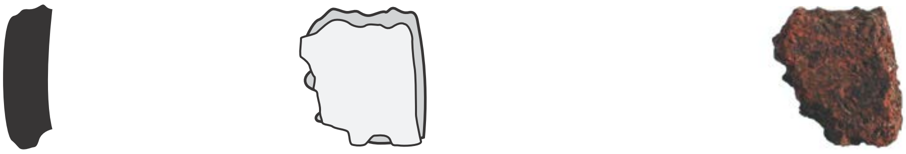

Wanelek pottery- fabric group 7

W11
plain
body

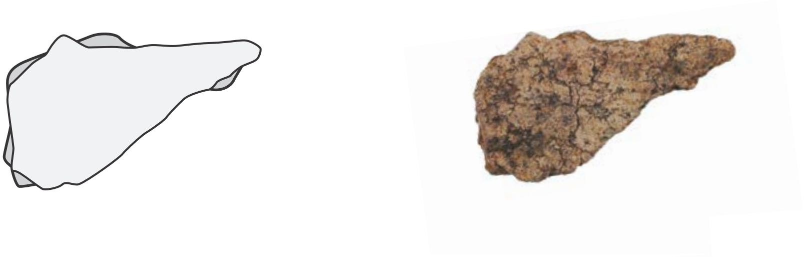

W12
plain
body

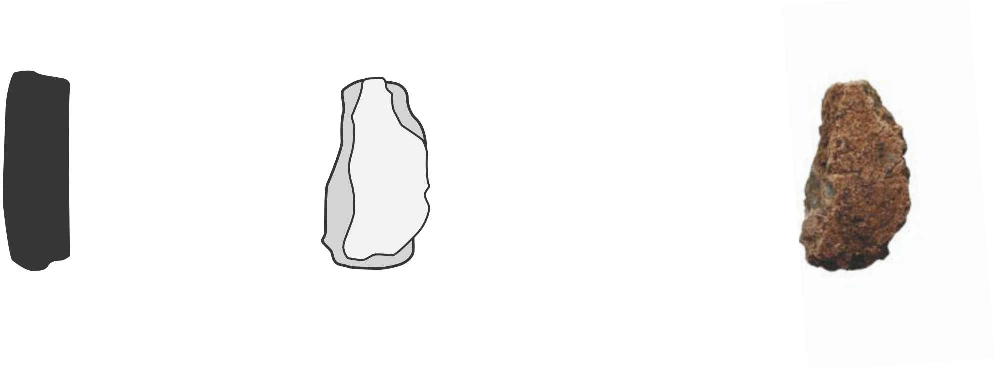

W14
plain
body

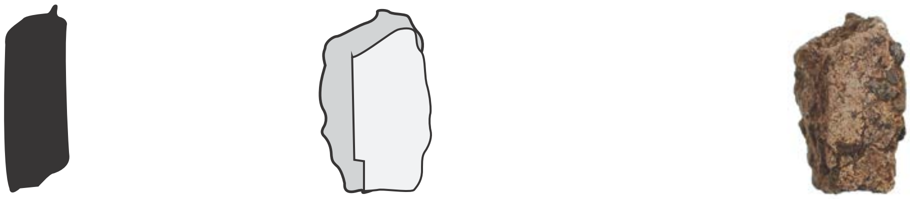

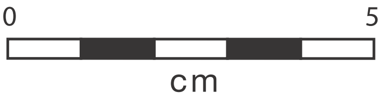

Supplement: S1 Appendix — (PDF) [file pone.0134497.s001.pdf]
