# Supplementary material for: Earliest Pottery on New Guinea Mainland Reveals Austronesian Influences in Highland Environments 3000 Years Ago
Source: PLoS One. 2015 Sep 2;10(9):e0134497. doi: 10.1371/journal.pone.0134497 (PMC4557931; doi:10.1371/journal.pone.0134497)

S.I. Appendix II

SEM micrographs of Wañelek pottery by fabric group

Non-plastic mineral inclusions

Fabric 1

W10

W10 mineral micrograph 1

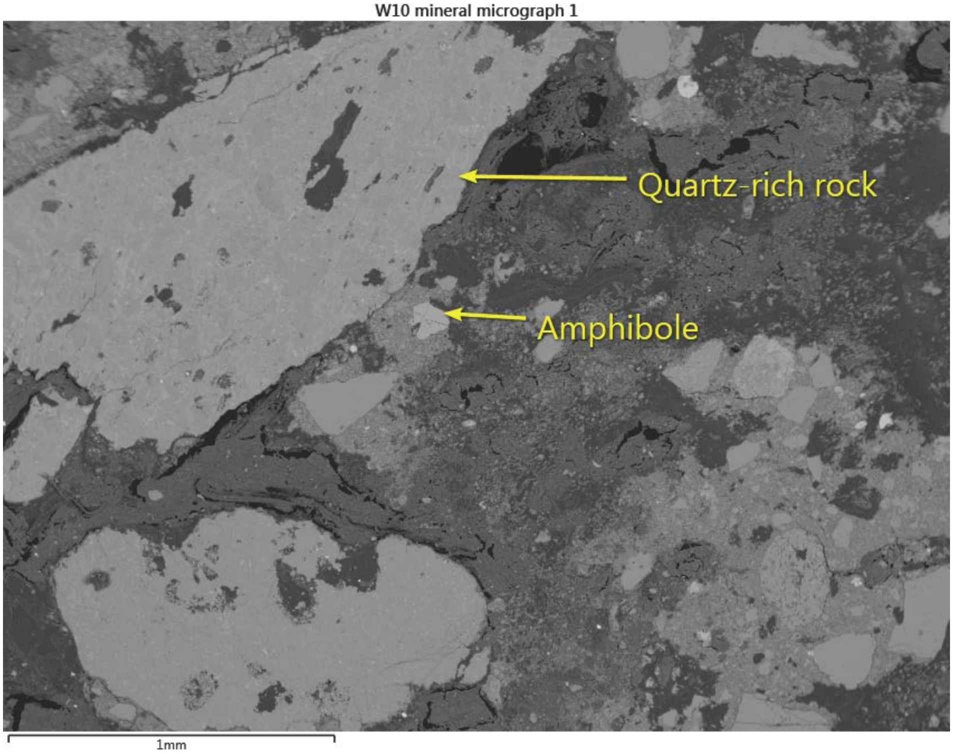

W10 mineral micrograph 2

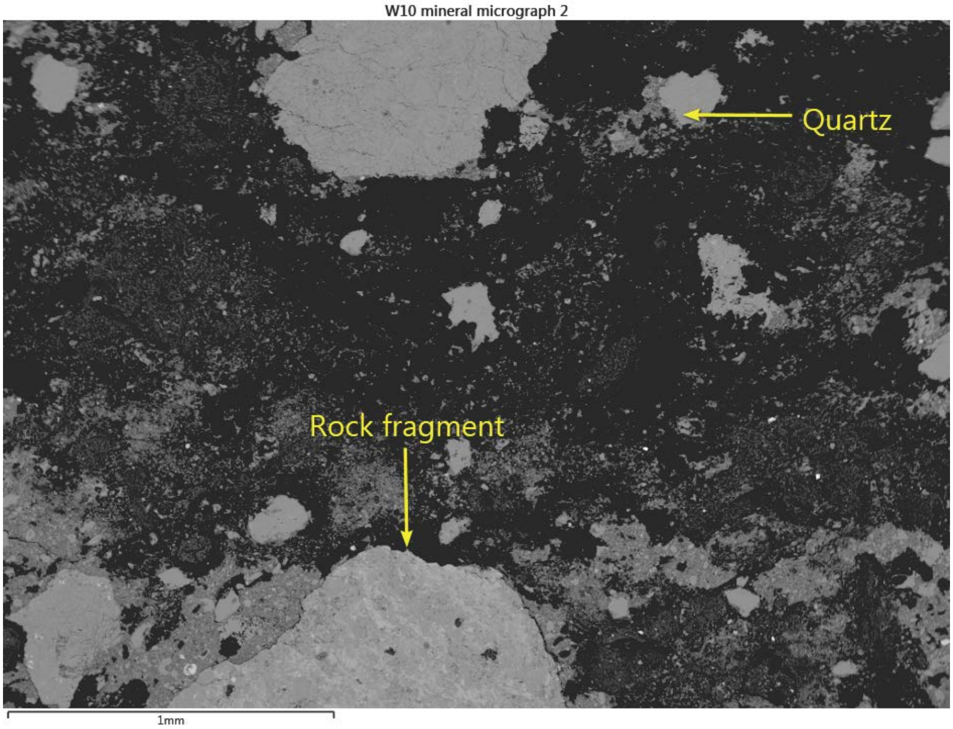

W10 mineral micrograph 3

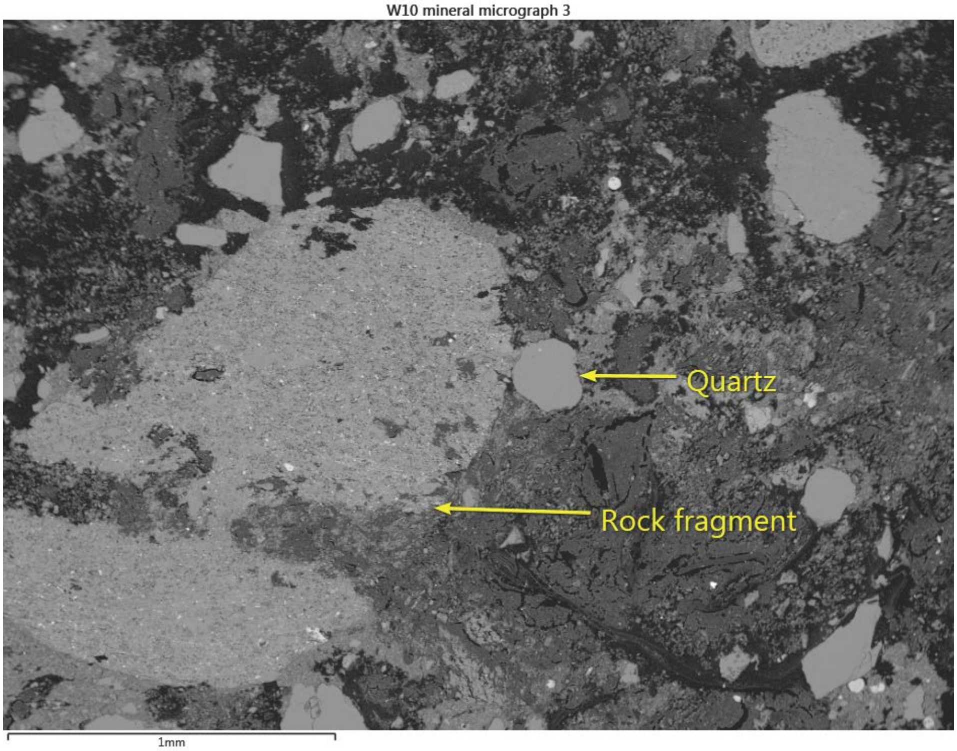

W16

W16 mineral micrograph 1

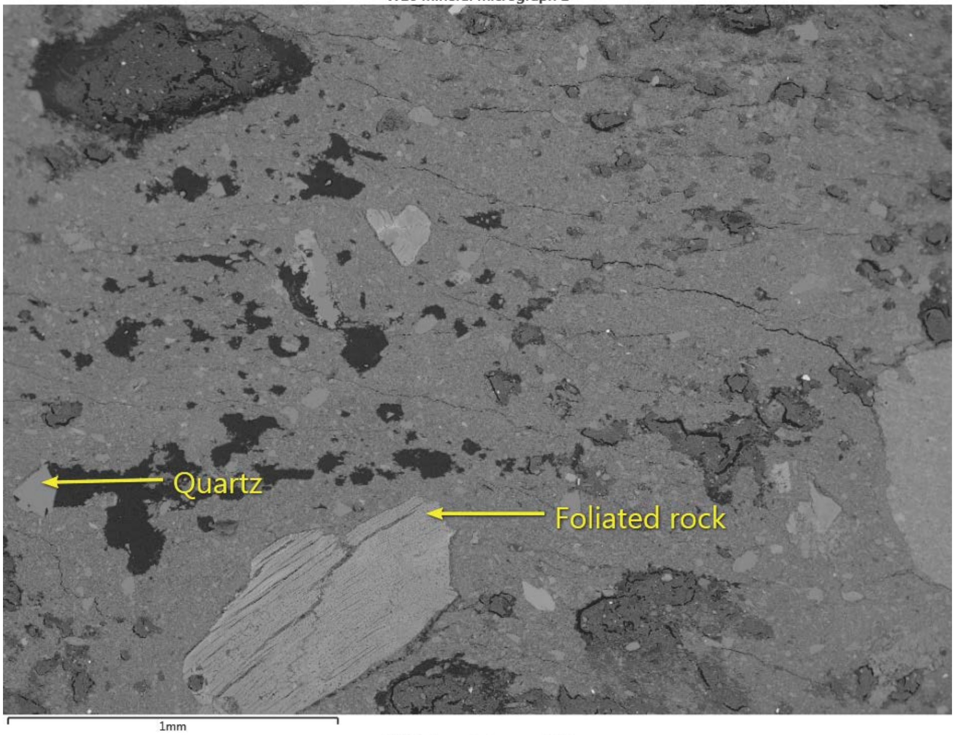

W16 mineral micrograph 2

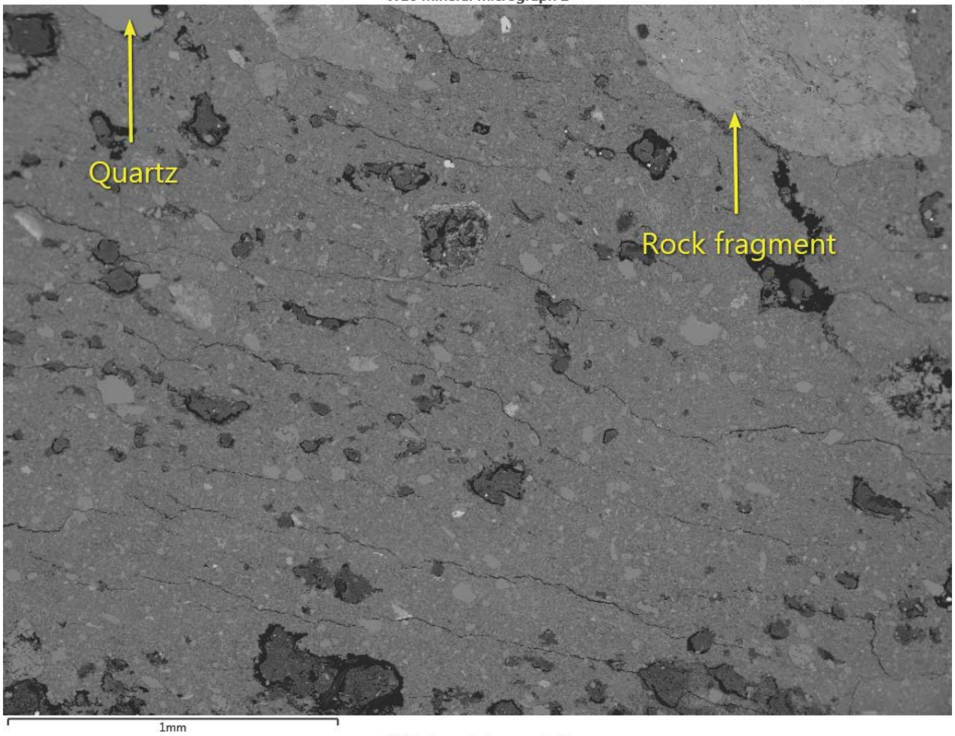

W16 mineral micrograph 3

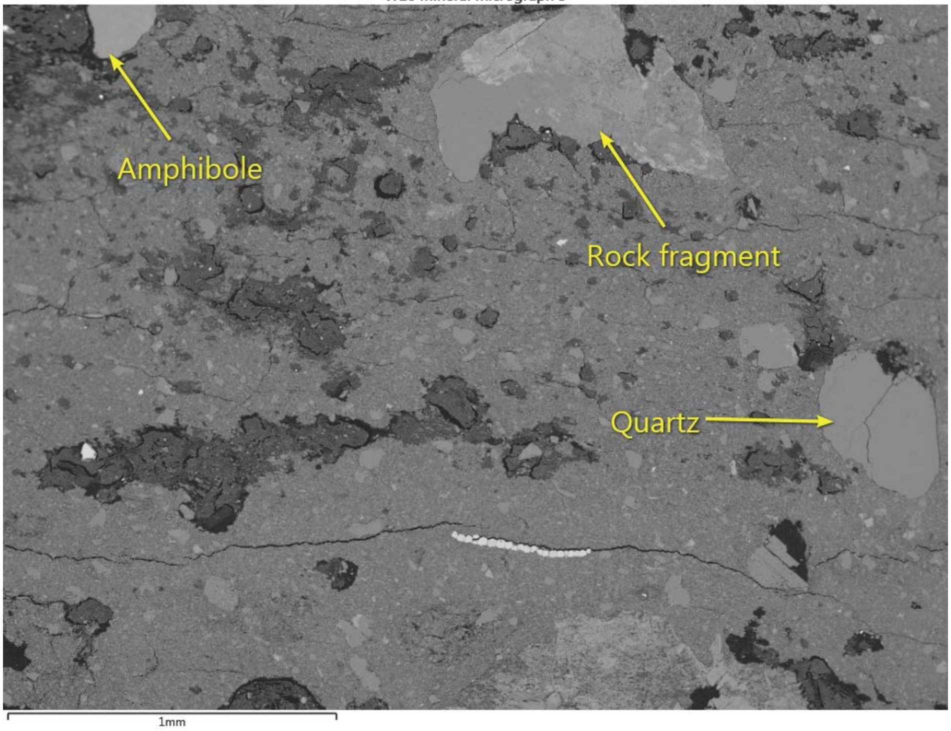

Fabric 2

W35

W35 mineral micrograph 1

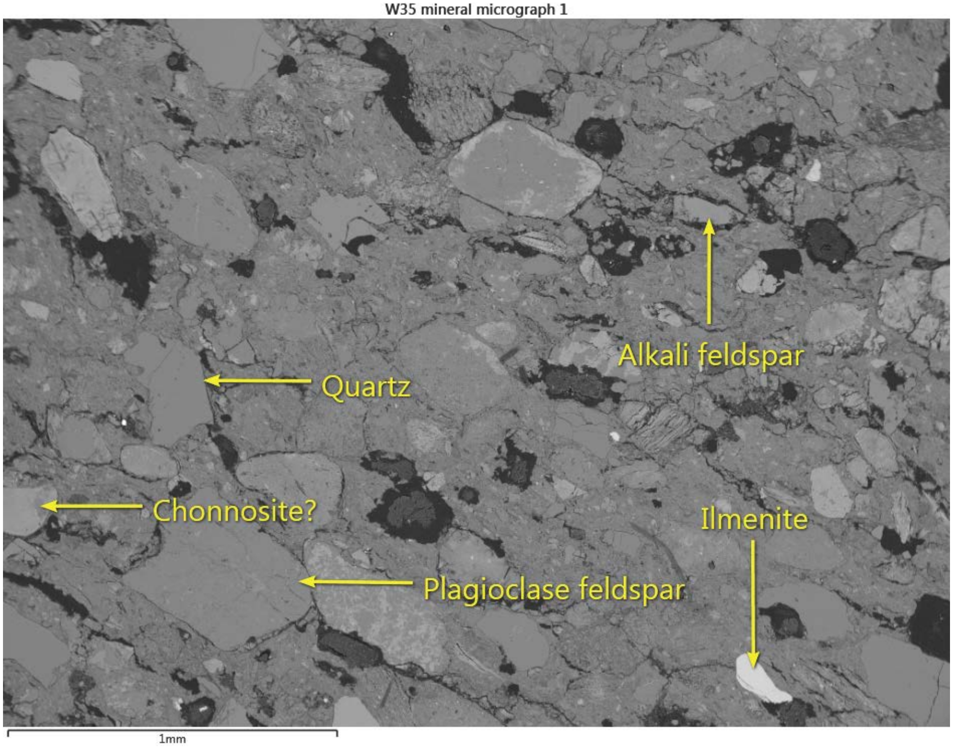

W35 mineral micrograph 2

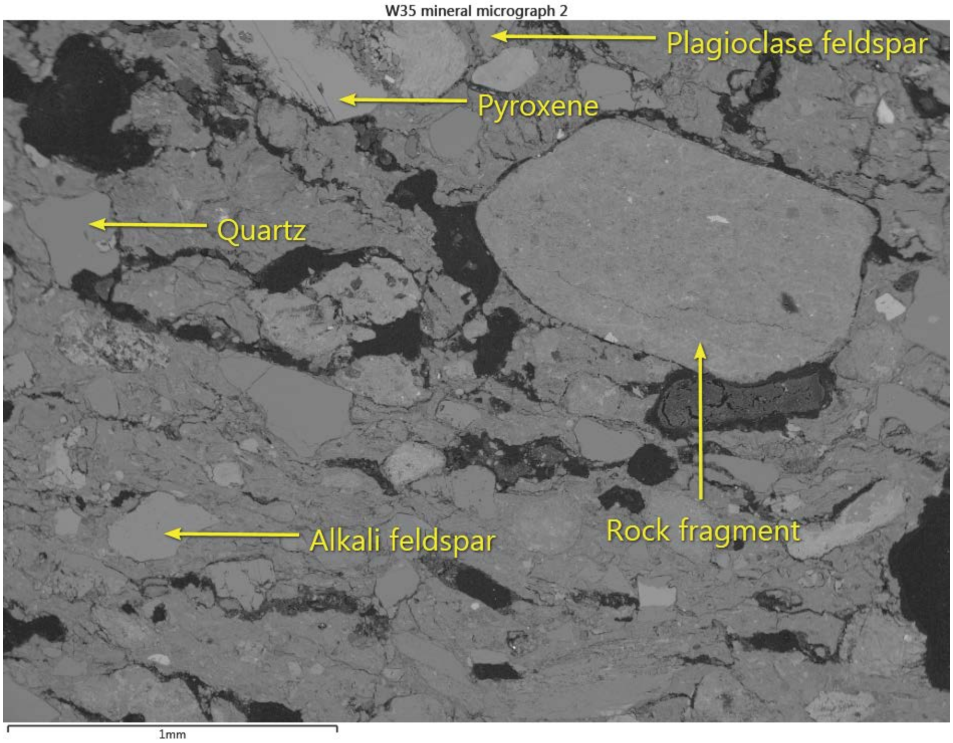

W35 mineral micrograph 3

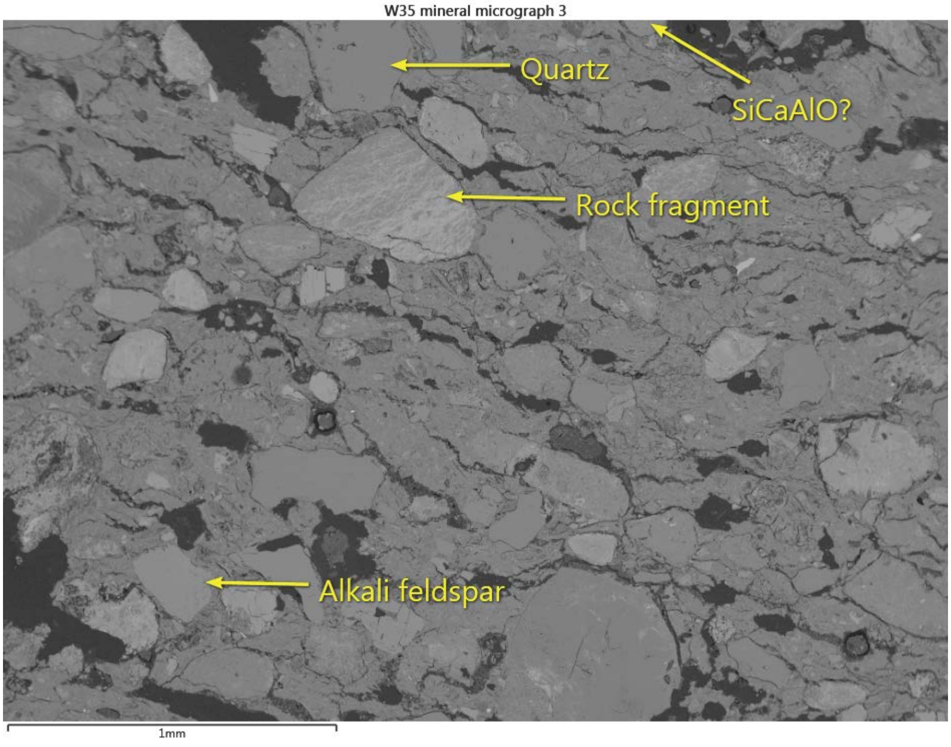

Fabric 3

W52

W52 mineral micrograph 1

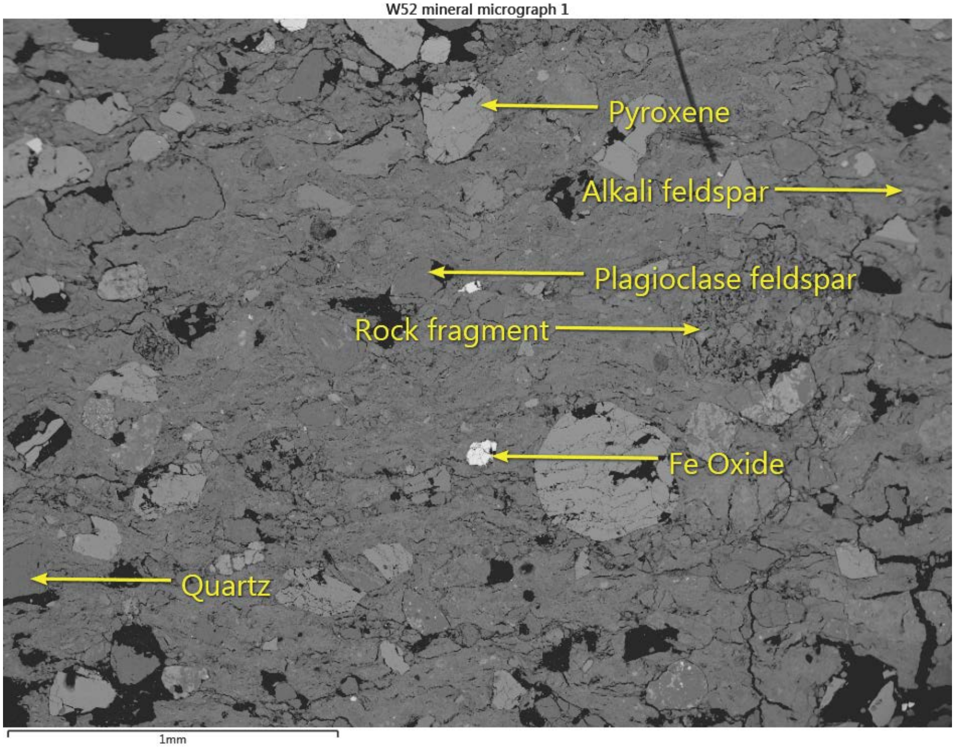

W52 mineral micrograph 2

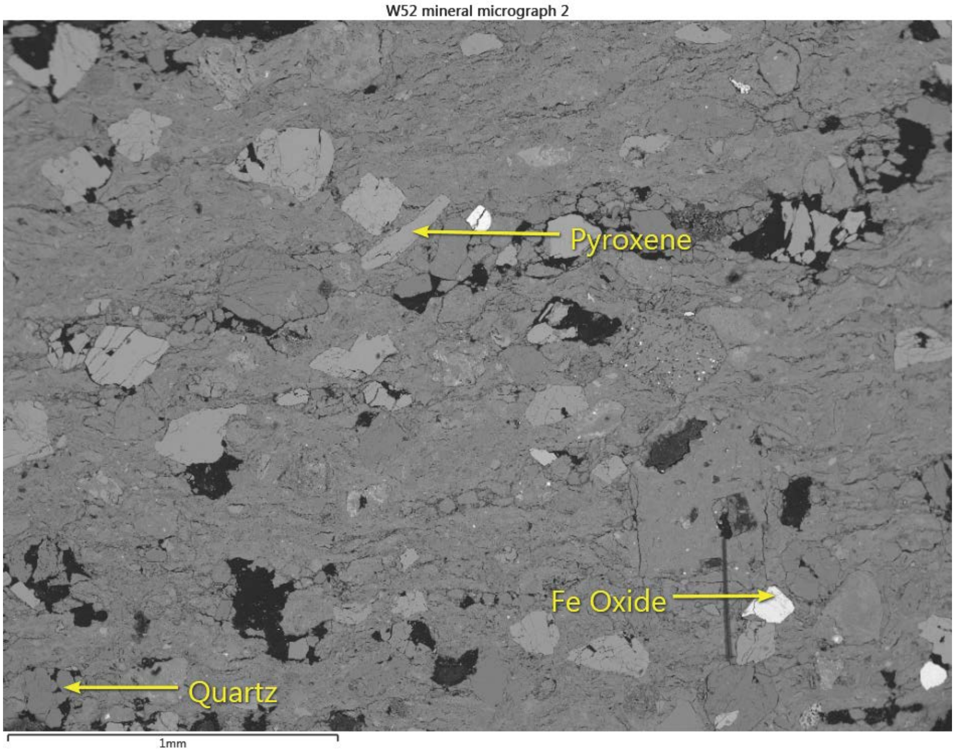

W52 mineral micrograph 3

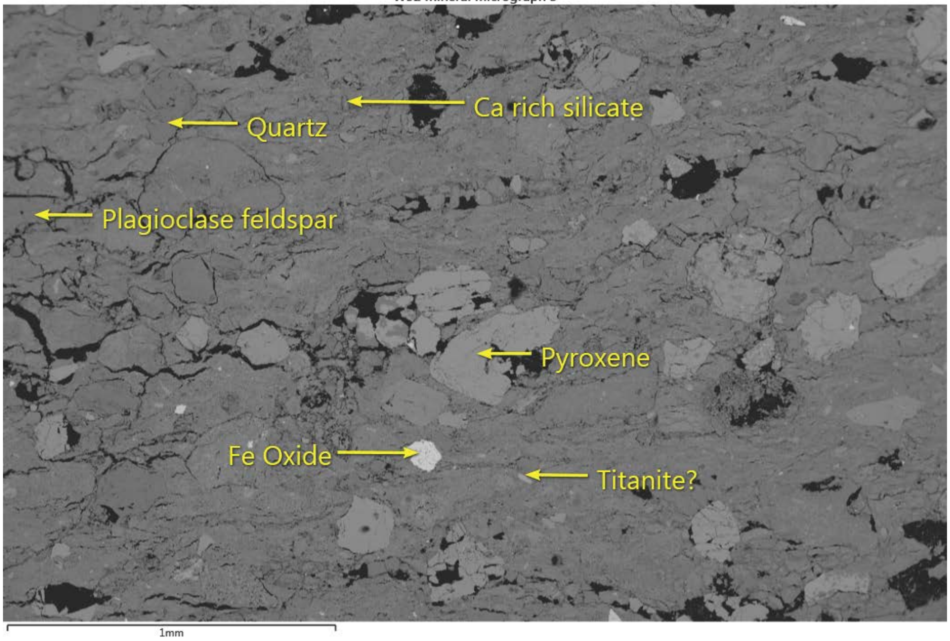

Fabric 4

W6

W6 minerals micrograph 1

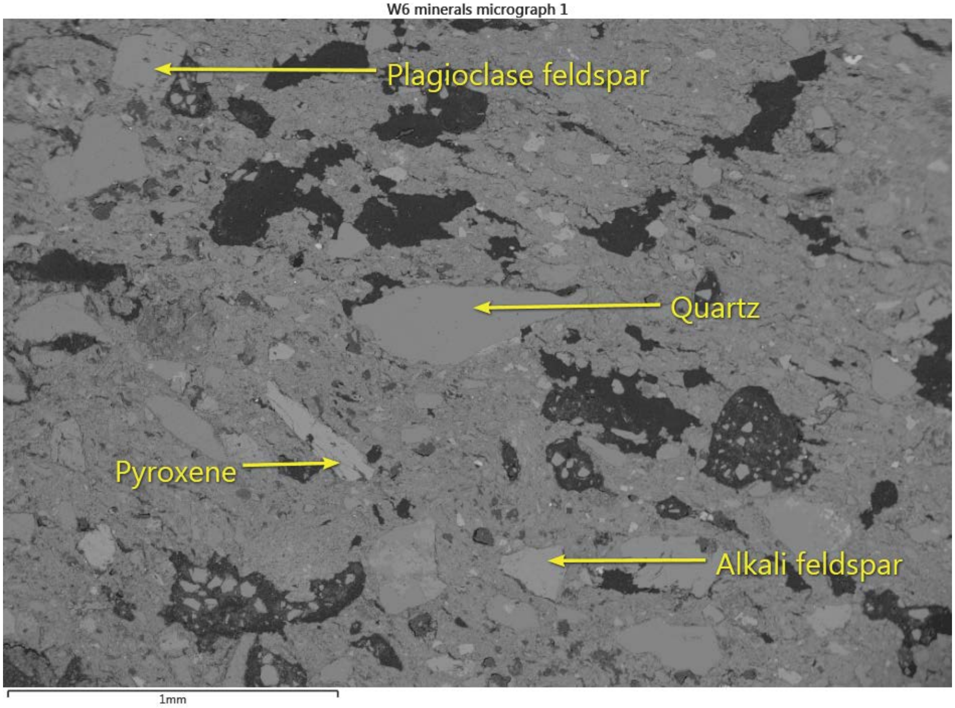

W6 minerals micrograph 2

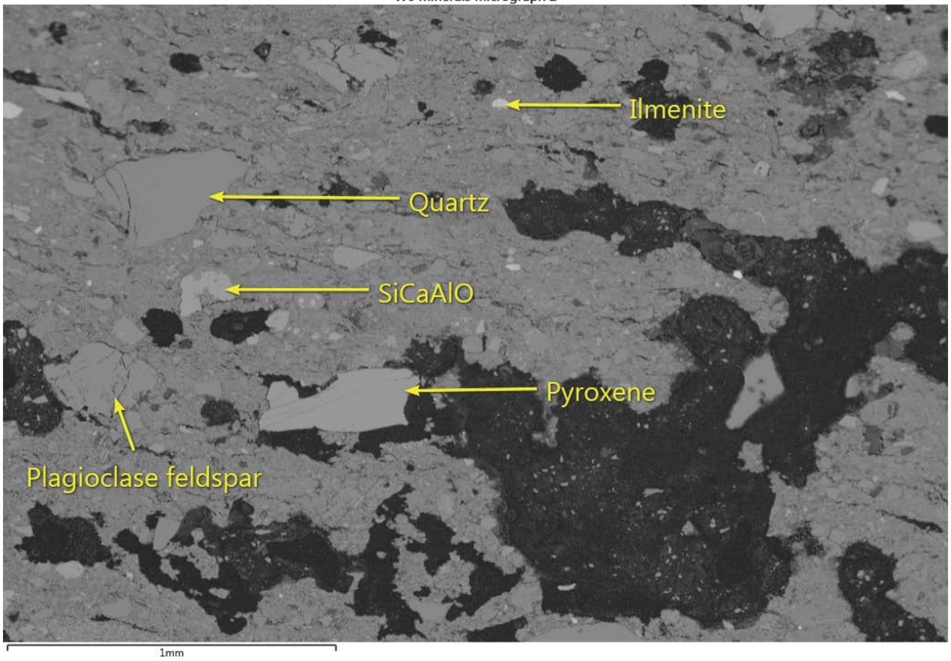

W6 minerals micrograph 3

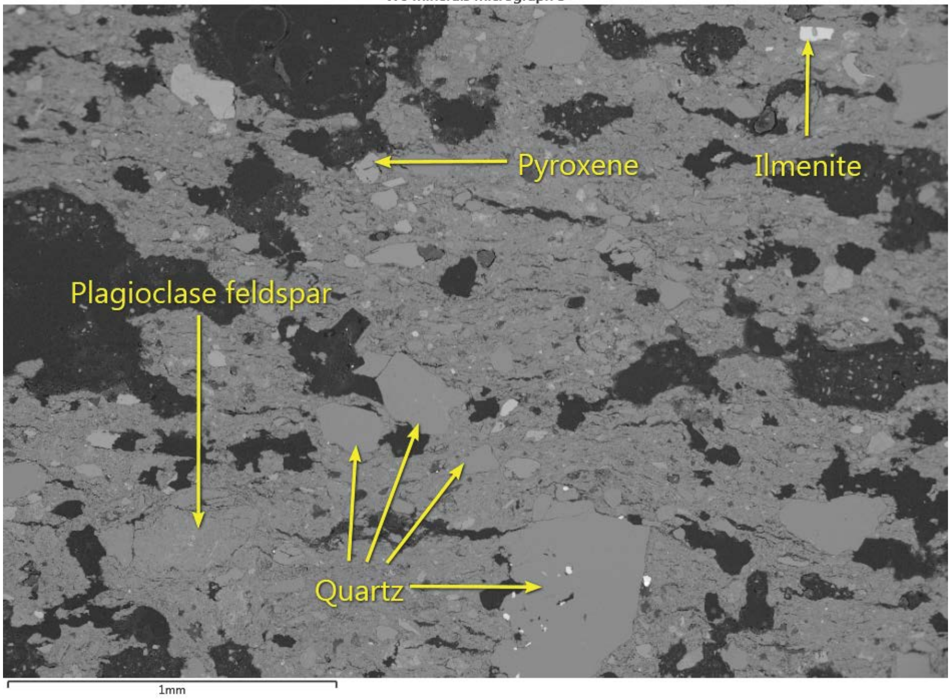

W9

W9 mineral micrograph 1

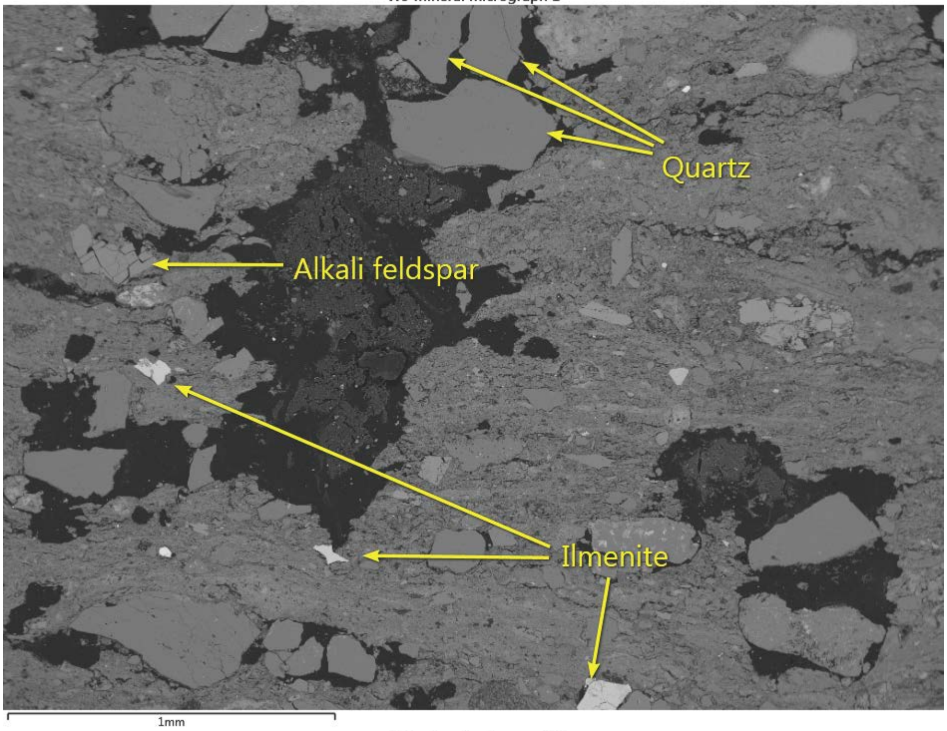

W9 mineral micrograph 2

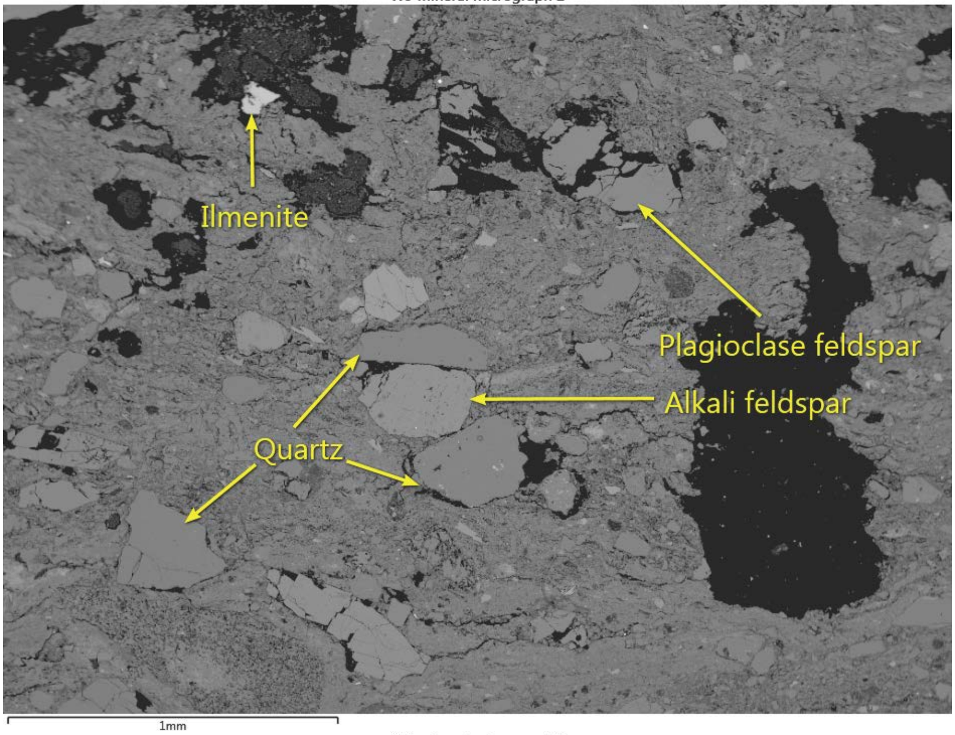

W9 mineral micrograph 3

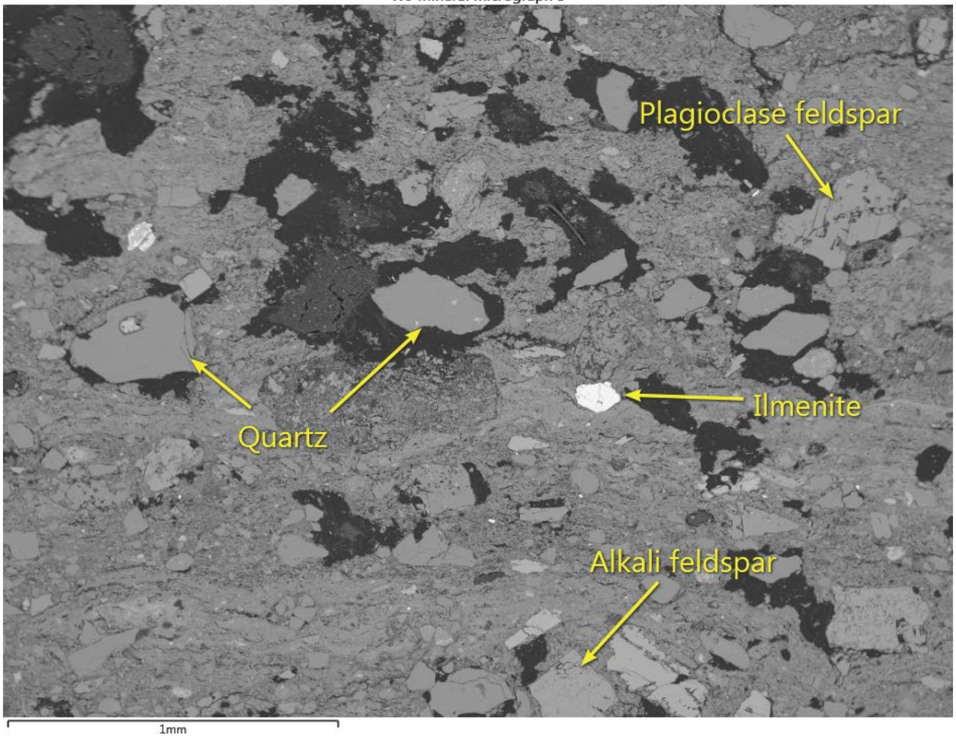

Fabric 5

W7

W7 mineral micrograph 1

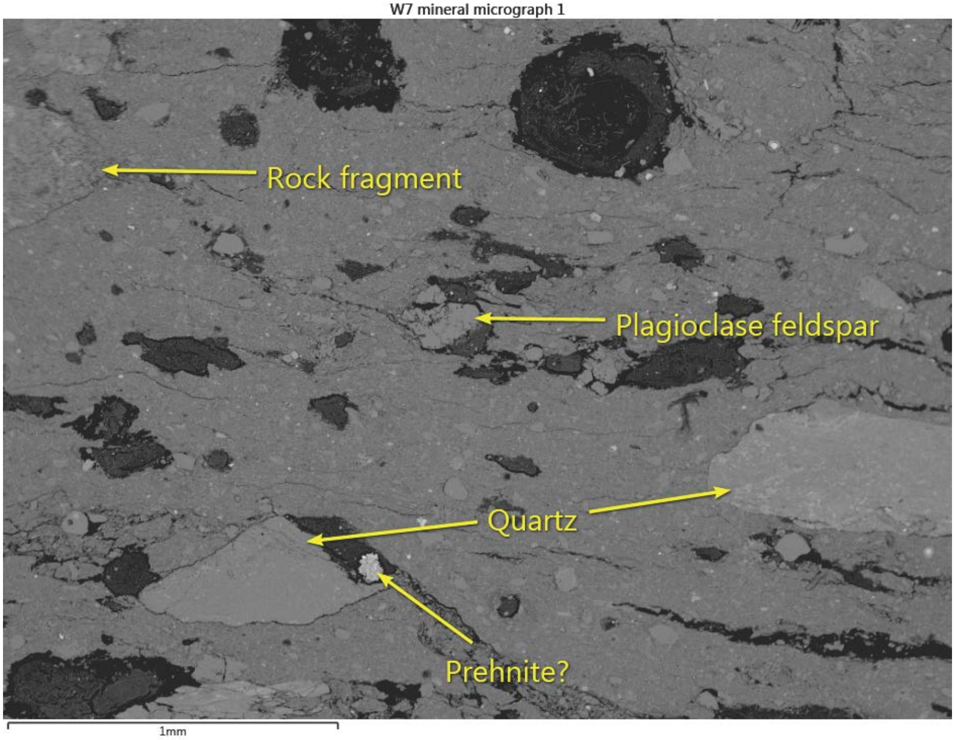

W7 mineral micrograph 2

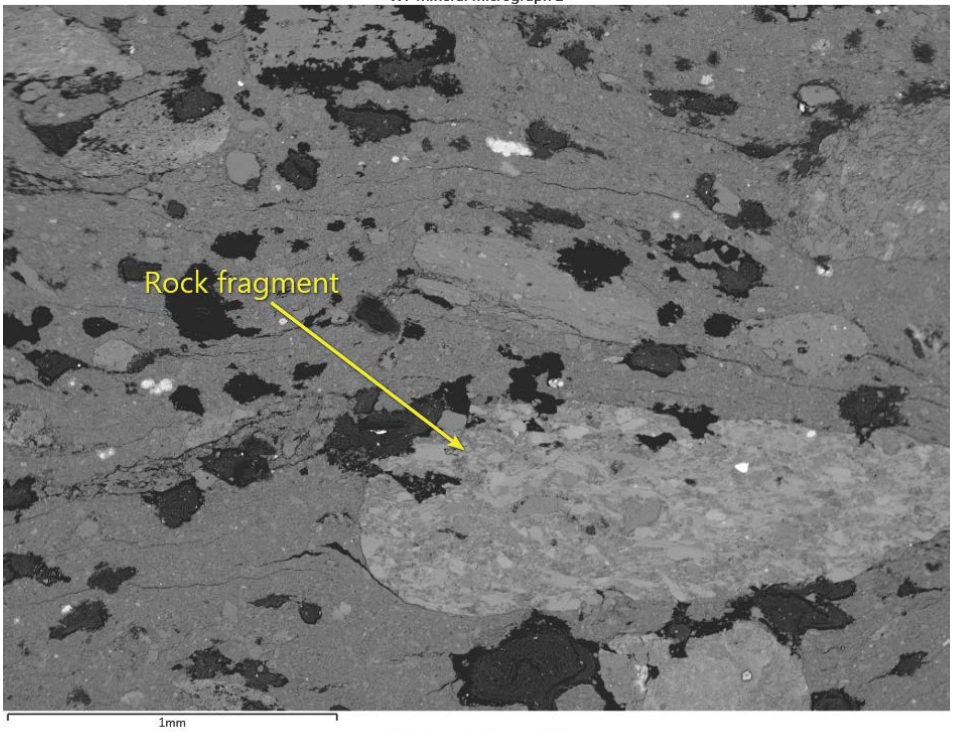

W7 mineral micrograph 3

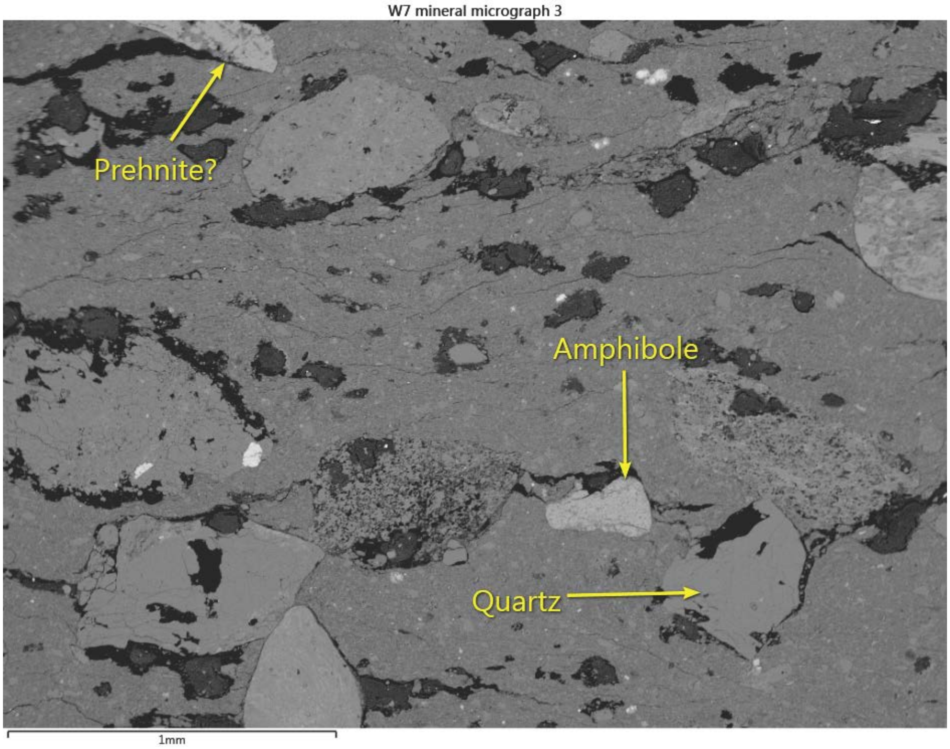

W45

W45 mineral micrograph 1

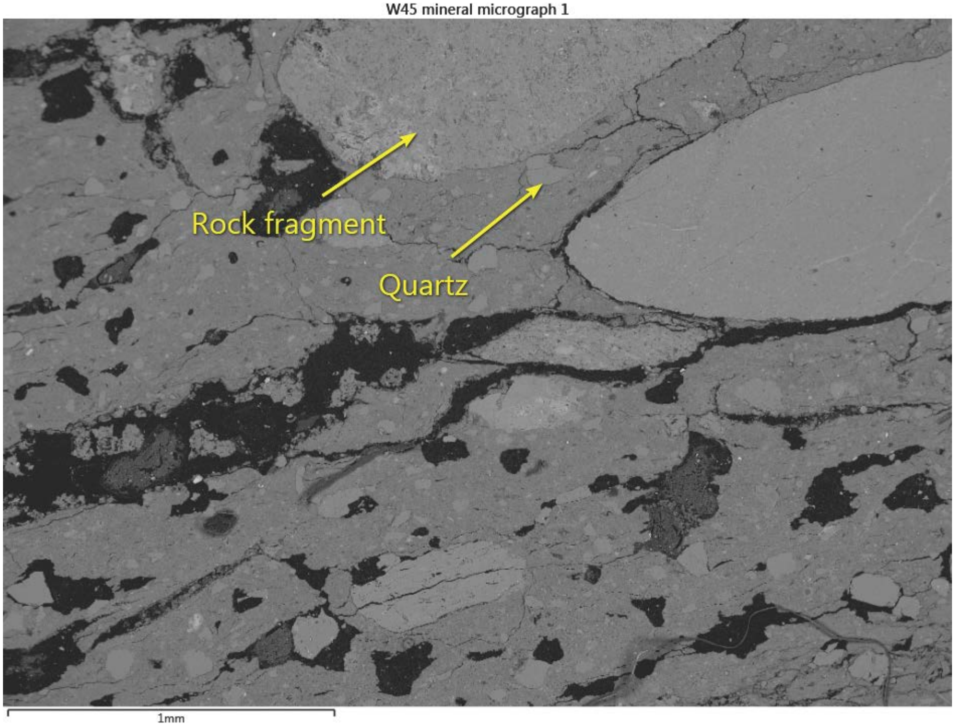

W45 mineral micrograph 2

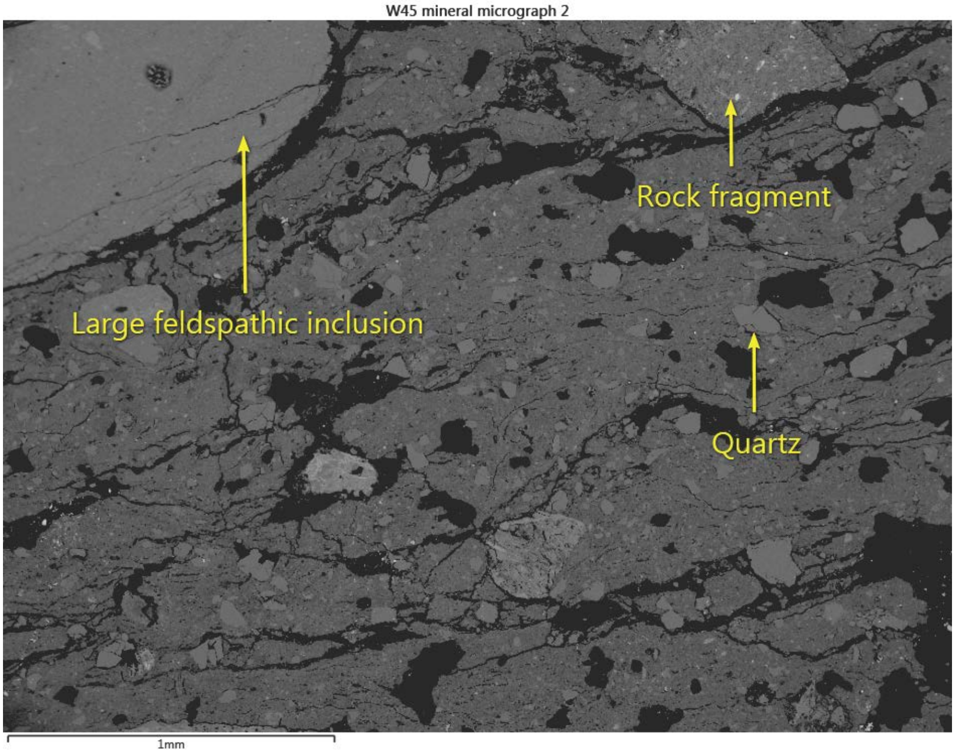

W45 mineral micrograph 3

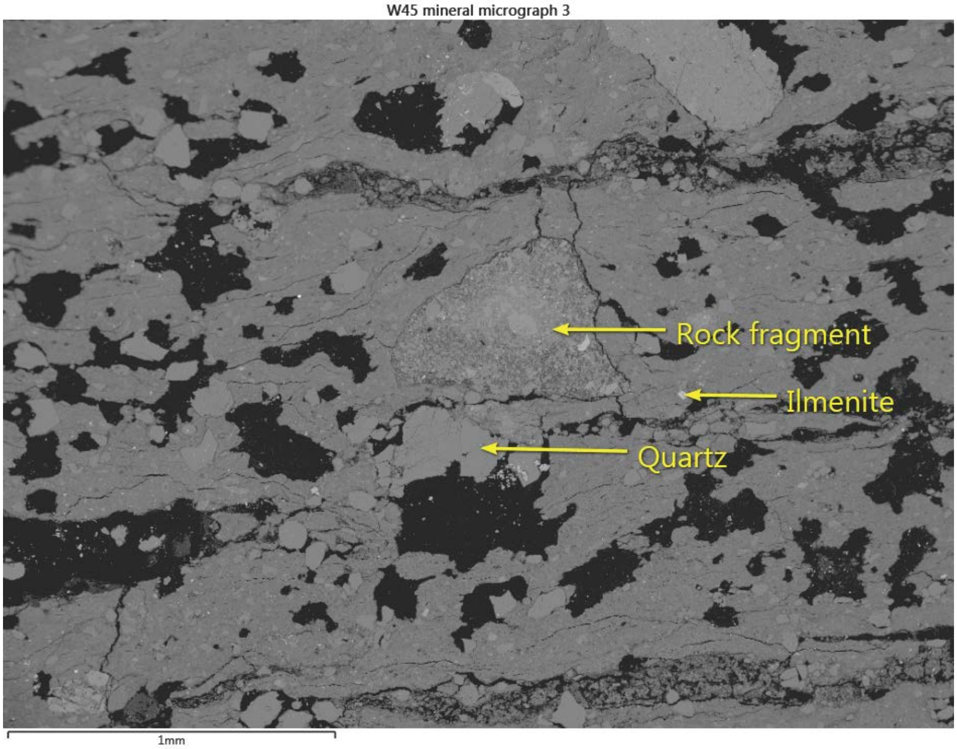

Fabric 6

W1

W1 minerals micrograph 1

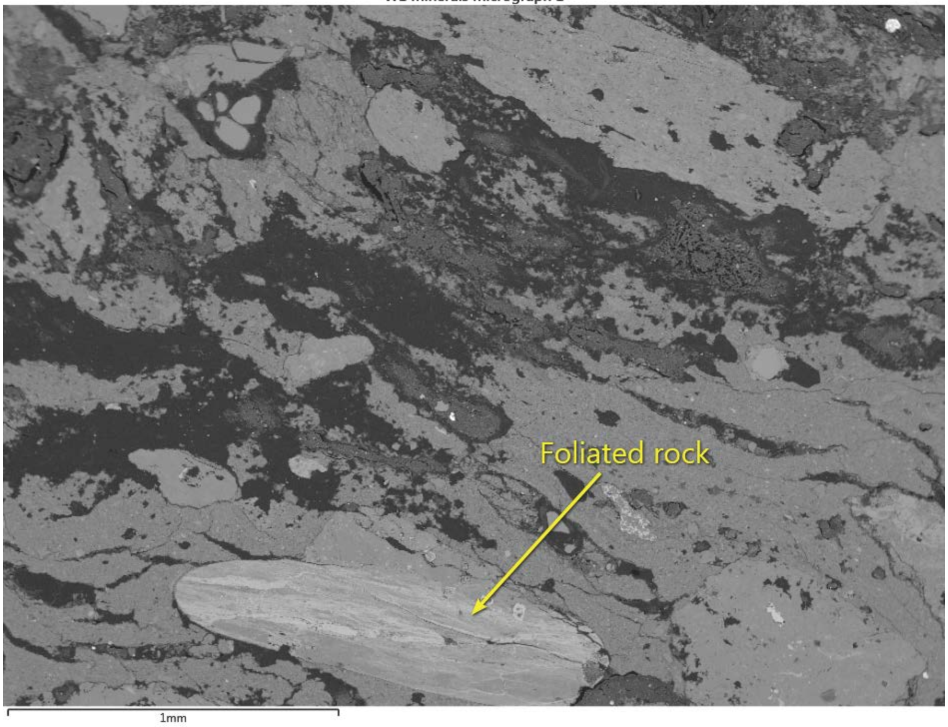

W1 minerals micrograph 2

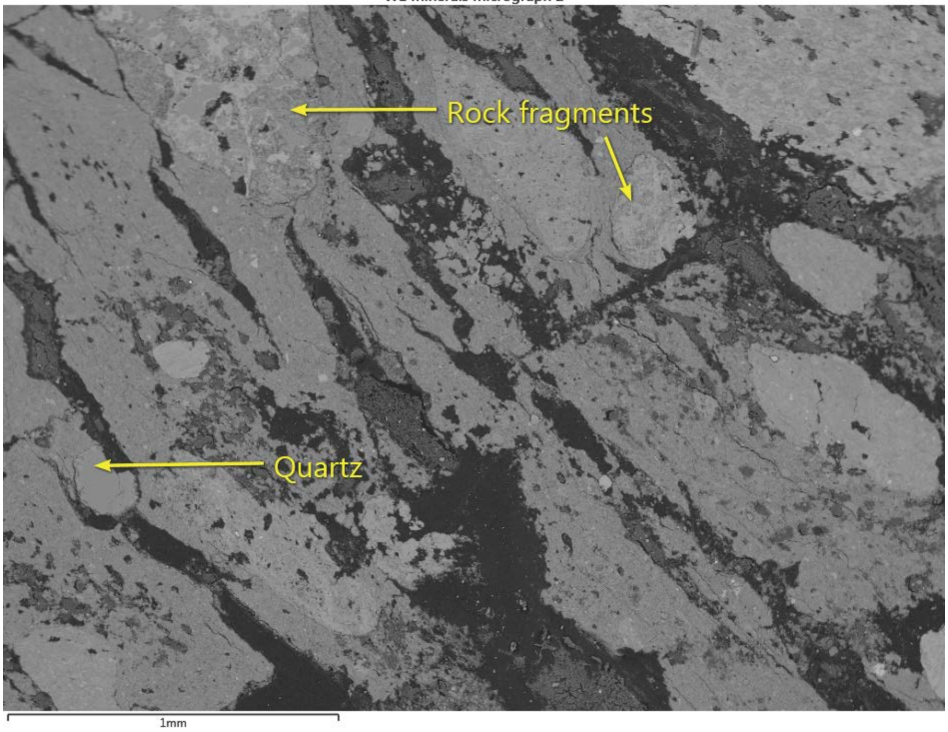

W1 mineral micrograph 3

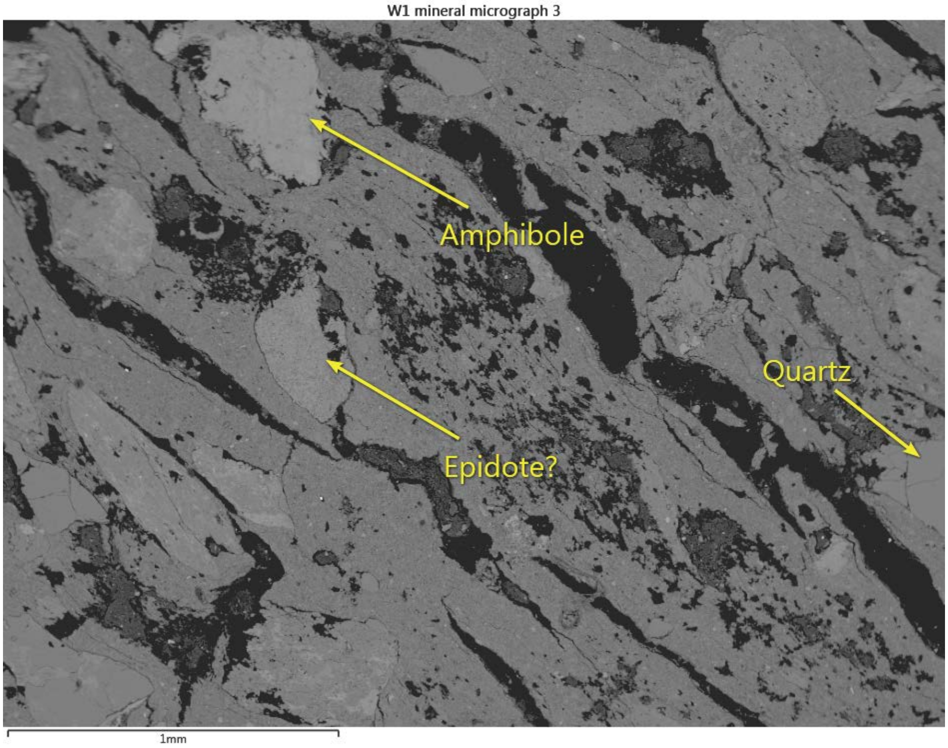

Fabric 7

W11

W11 mineral micrograph 1

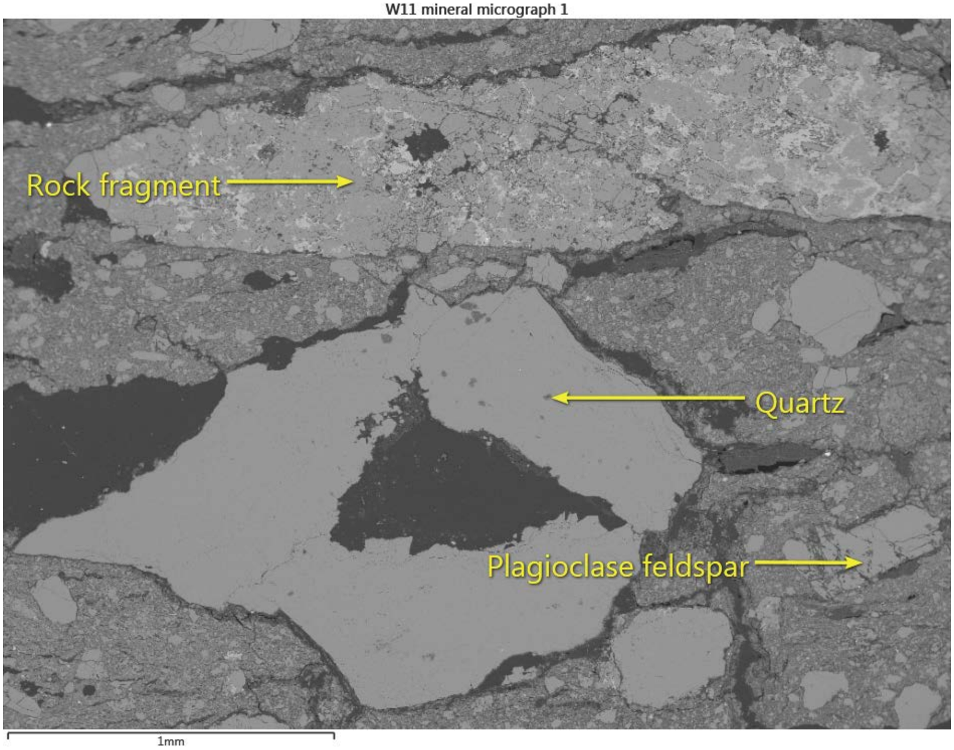

W11 mineral micrograph 2

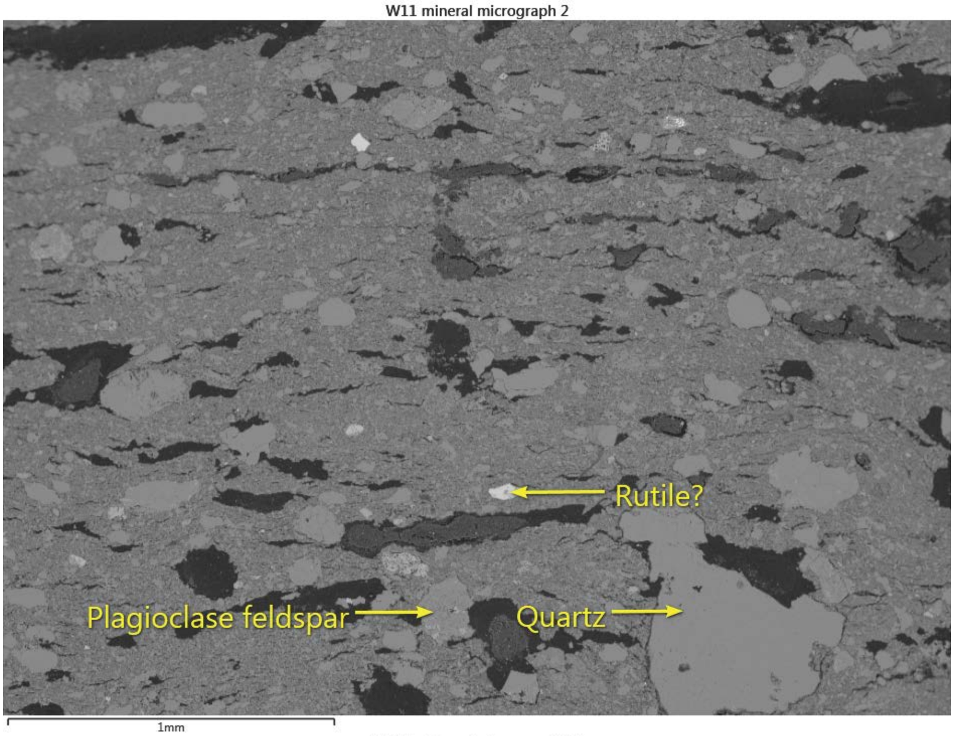

W11 mineral micrograph 3

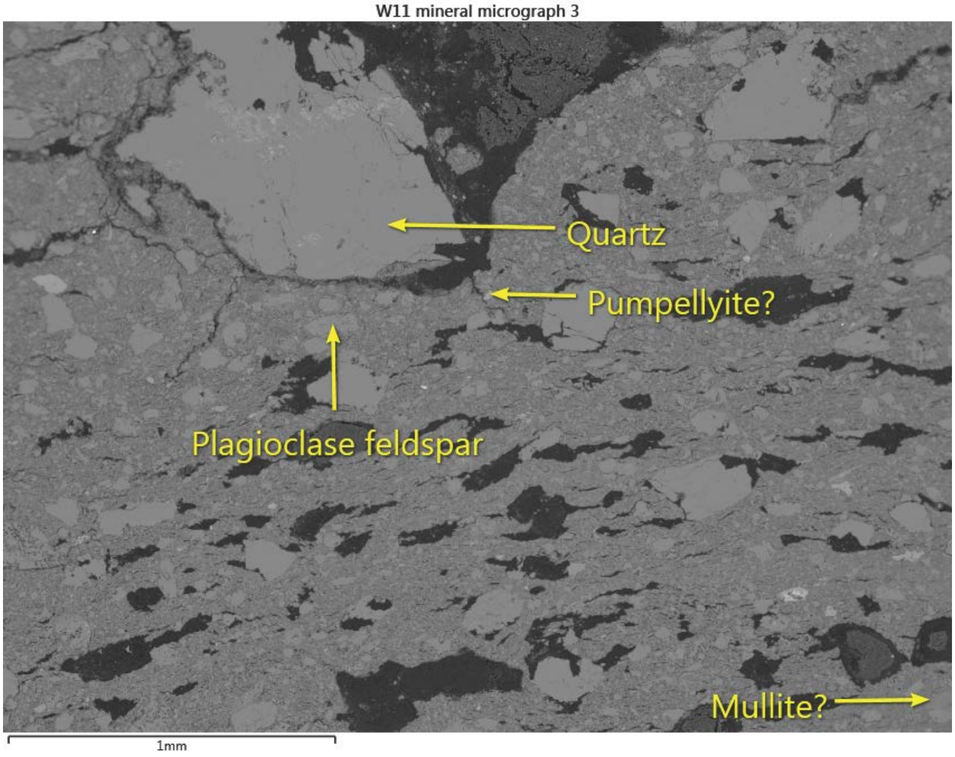

W12

W12 mineral micrograph 1

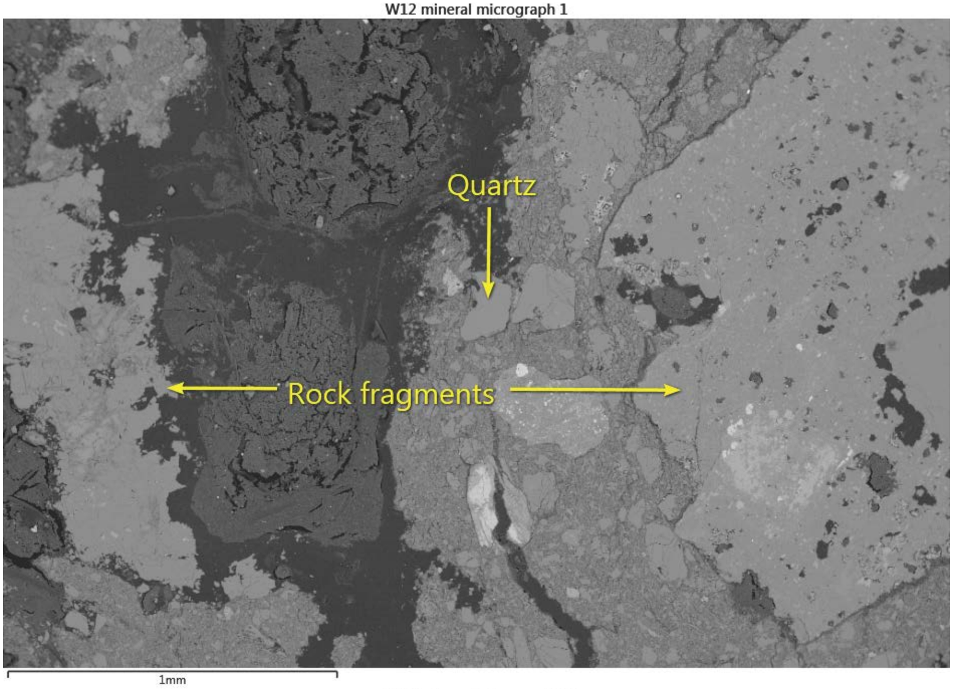

W12 mineral micrograph 2

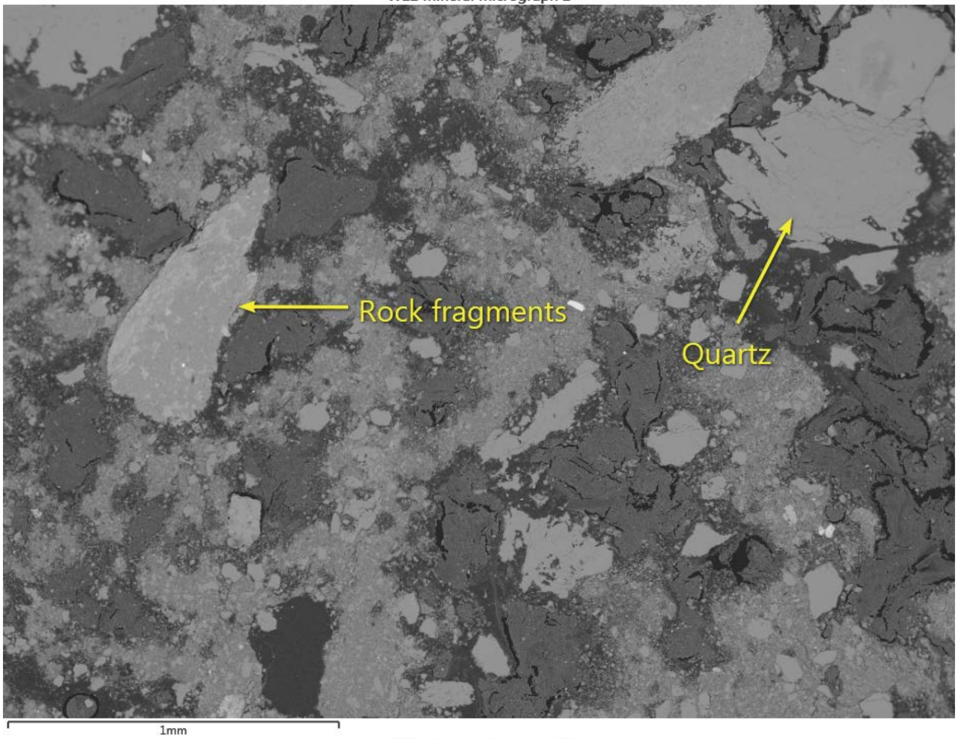

W12 mineral micrograph 3

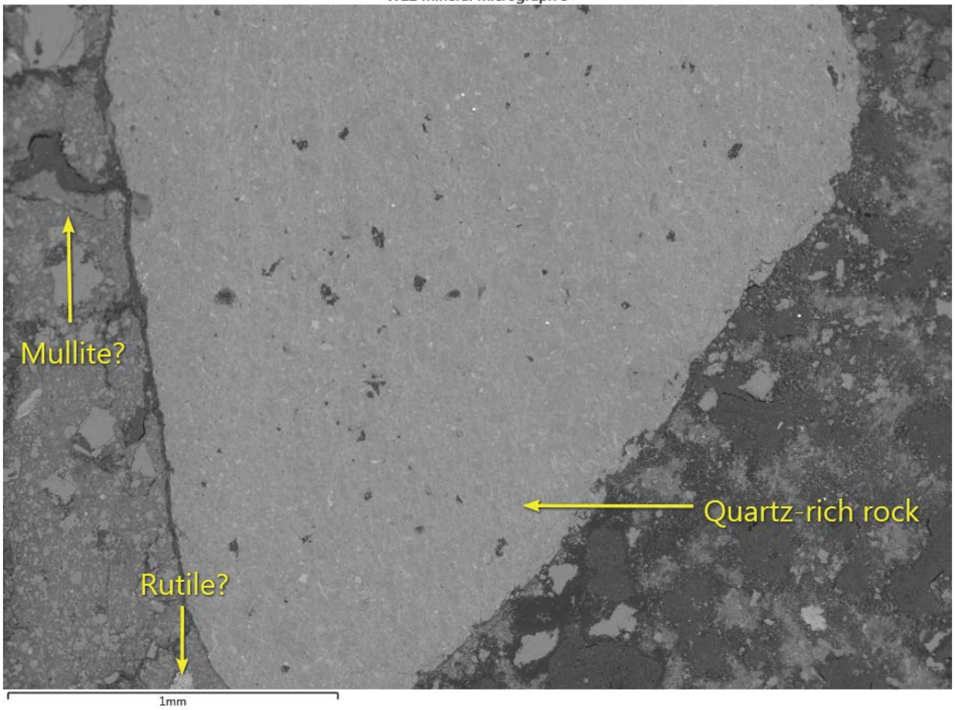

W14

W14 mineral micrograph 1

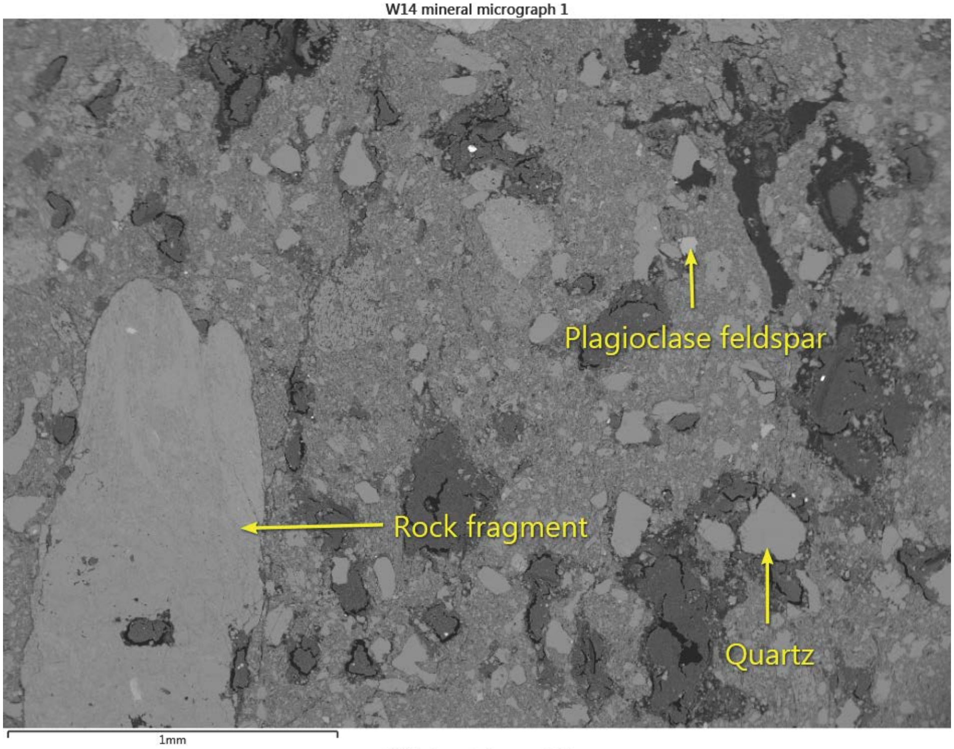

W14 mineral micrograph 2

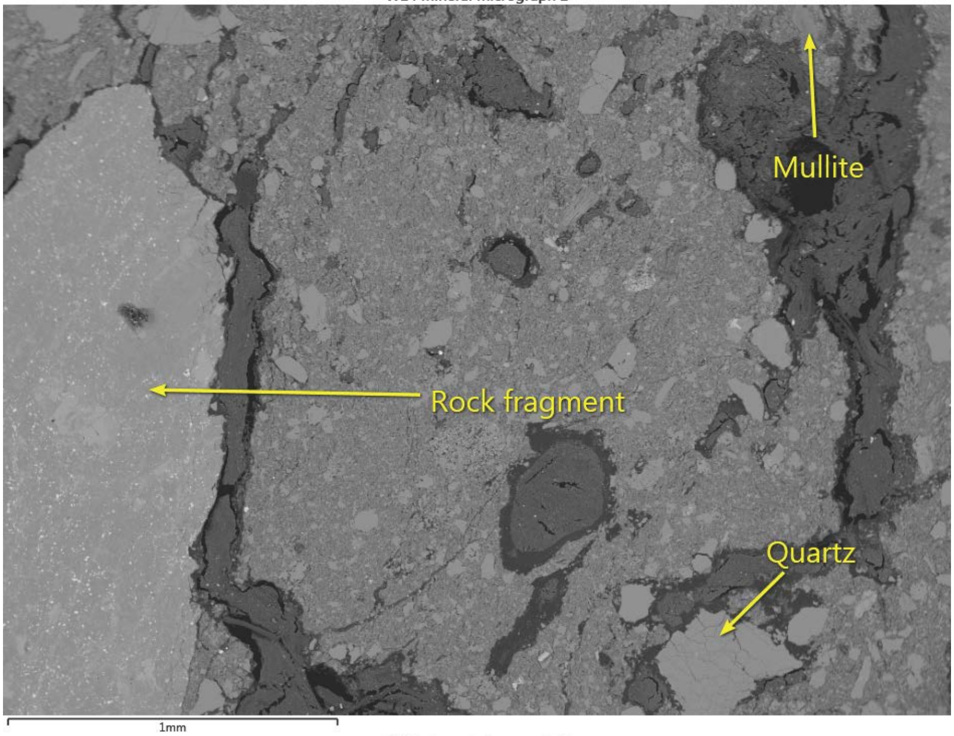

W14 mineral micrograph 3

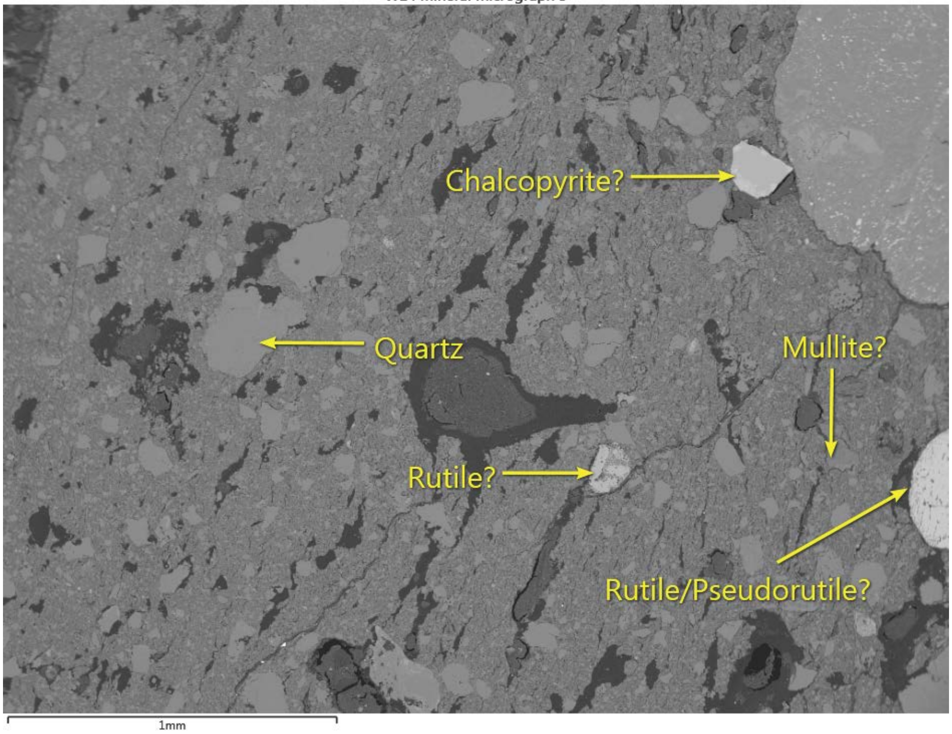

Supplement: S2 Appendix — (PDF) [file pone.0134497.s002.pdf]
