# Supplementary material for: Earliest Pottery on New Guinea Mainland Reveals Austronesian Influences in Highland Environments 3000 Years Ago
Source: PLoS One. 2015 Sep 2;10(9):e0134497. doi: 10.1371/journal.pone.0134497 (PMC4557931; doi:10.1371/journal.pone.0134497)
Supplement: S1 Table — Highlighted samples were sampled for SEM analysis. (DOCX) [file pone.0134497.s004.docx]

Table S1. Summary of macroscopic fabric groups and associated mineralogies by map scanning. Highlighted samples were sampled for SEM analysis.

| Fabric group | Samples | Clay matrix (%) | Mineral inclusions (%) |
| --- | --- | --- | --- |
| 1 | W3  W10  W16  W50  W55 | Clay (79%) | Quartz (2%)  Plagioclase feldspar (2%)  Rock fragments (hornblendes)  Alkali feldspar (<1%)  Amphibole (<1%)  Haematite (<1%)  Rutile (<1%) |
| 2 | W2  W4  W5  W13  W54  W35 | Clay (57%) | Quartz (12%)  Plagioclase feldspar (oligoclase?) (14%)  Andalusite (or another Kyanite?) (1%)  Alkali feldspar (1%)  Pyroxene (Ferroan augite) (1%)  Chonnosite? (Fe rich silicate) (1%)  Ilmenite (<1%)  SiCaAlO? (<1%) |
| 3 | W52 | Clay (71%) | Quartz (8%)  Pyroxene (augite) (8%)  Amphibole (3%)  Plagioclase feldspar (albite?) (3%)  Alkali feldspar (<1%)  Chegmite? (Ca rich silicate) (<1%)  Fe-Oxide (<1%)  Titanite? (<1%) |
| 4 | W6  W9 | Clay (56%) | Quartz (16%)  Plagioclase feldspar (oligolcase?) (3%)  Alkali feldspar (orthoclase?) (2%)  Pyroxene (1%)  Andalusite (<1%)  Ilmenite (<1%)  Olivine? (<1%)  SiCaAlO? (<1%) |
| 5 | W7  W45 | Clay (69%) | Quartz (8%)  Feldspathic rock fragments (present)  Amphibole (<1%)  Haematite (<1%)  Ilmenite (<1%)  Prehnite (or pumpellyite/porphyrite?) (<1%) |
| 6 | W1 | Clay (68%) | Quartz (2%)  Rock (foliated hornblende) (present)  Olivine? (3%)  Amphibole (2%)  SiCaAlO? (2%)  Staurolite? (<1%) |
| 7 | W11  W12  W14 | Clay (52%) | Quartz (17%)  Plagioclase feldspar (9%)  Rock fragments (present)  Mullite (<1%)  Rutile (<1%)  Fe-Oxide (<1%)  Chalcopyrite? (<1%)  Pumpellyite? (<1%) |
