# Supplementary material for: Earliest Pottery on New Guinea Mainland Reveals Austronesian Influences in Highland Environments 3000 Years Ago
Source: PLoS One. 2015 Sep 2;10(9):e0134497. doi: 10.1371/journal.pone.0134497 (PMC4557931; doi:10.1371/journal.pone.0134497)
Supplement: S2 Table — (DOCX) [file pone.0134497.s005.docx]

Table S2. Estimates for earliest dates of pottery fabric groups at Wañelek

| Fabric | Sherds | Excavation unit | Excavated layer | Associated date |
| --- | --- | --- | --- | --- |
| 1 | W3 | Unstratified | Surface | Unknown |
|  | W16 | 11B | Layer 8 | Younger than 3840BP? |
|  | W10  W55  W50 | 101Y | Layer 2 | Younger than 872BP? |
|  |  |  | Layer 3 | ~872BP |
| 2 | W2  W4  W5 | Unstratified | Surface | Unknown |
|  | W13  W35 | 105Z | Layer 5 | ~3697BP |
|  |  |  | Layer 7 | Older than 3697BP? |
|  | W54 | 10C | Layer 5 | Younger than 3840BP? |
| 3 | W52 | TR1 | Layer 5 (Pit feature) | Older than 2840BP. ~2865BP. |
| 4 | W6 | 17/18B(II) | Layer 2 | Younger than 3052BP? |
|  | W9 | 101Z | Layer 5 | ~3697BP? |
| 5 | W7 | 17/18B(II) | Layer 2 | Younger than 3052BP? |
|  | W45 | 16B | Layer 6 | ~3225BP |
| 6 | W1 | Unstratified | Surface | Unknown |
| 7 | W11  W12  W14 | 105Z | Layer 5 | ~3697BP |
