# Supplementary material for: Earliest Pottery on New Guinea Mainland Reveals Austronesian Influences in Highland Environments 3000 Years Ago
Source: PLoS One. 2015 Sep 2;10(9):e0134497. doi: 10.1371/journal.pone.0134497 (PMC4557931; doi:10.1371/journal.pone.0134497)
Supplement: S3 Table — (DOCX) [file pone.0134497.s006.docx]

Table S3. Comparative fabric reports on modern pottery from the New Guinea Highlands and nearby areas.

| Manufacture | Clay type | Inclusions | Reference |
| --- | --- | --- | --- |
| Upper Ramu headwaters | Lean clay | Poorly sorted gneissic minerals, mostly quartz and feldspar. Some dark green hornblende. | Key 1973* |
| Agarabi (Kainantu) | Lean clay | Poorly sorted quartz (25%), weathered K-Na feldspars (10.4%), granite and quartzite rock fragments (5.2%), and occasional green hornblende. | Key 1969 (Appendix 3 in White 1972:160-161). Pot collected by Coutts (1967:482) |
| Agarabi (Bilimoia Village) | Dark brown | Quartz and K feldspar and some plagioclase | Goodspeed (Appendix 5, in Watson and Cole 1977: 198) |
| Usur (Hills region) | - | Feldspars, quartz, weathered basalts. Less than 5% inclusions. | Hughes 1977 |
| Usur (Sumau) | Grey yellowish brown smectite clay (18%) | Non-plastics (82%)- including iron stained quartose sand, some igneous rock fragments | May and Tuckson 2000 |
| Keram River | - | ‘Clay contains much grit and rubble’ | May and Tuckson 2000 |
| Rao | Brownish grey clay | ‘Free of rubble but sandy’ | May and Tuckson 2000 |
| Finnisterre Ranges (southern fringes) | - | Poorly sorted feldspar, pyroxene, and basalt, without shell | Key 1969 (Appendix 3 in White 1972:160-161) citing Schmitz (1960:81) |

*Key, C. A. (1973). Pottery Manufacturing Techniques in Papua-New Guinea. *Asian Perspectives* 14: 65-68.
