# Supplementary material for: Earliest Pottery on New Guinea Mainland Reveals Austronesian Influences in Highland Environments 3000 Years Ago
Source: PLoS One. 2015 Sep 2;10(9):e0134497. doi: 10.1371/journal.pone.0134497 (PMC4557931; doi:10.1371/journal.pone.0134497)
Supplement: S4 Table — (DOCX) [file pone.0134497.s007.docx]

Table S4. Archaeological pottery from excavated New Guinea Highland sites

| Site | Code | Type | Province | No. of sherds | Reference |
| --- | --- | --- | --- | --- | --- |
| Wañelek | JAO | Open | Madang | 20* | Bulmer 1985 |
| Aibura | NAE | Cave | Eastern Highlands | 16 | White 1972 |
| Kiowa | NAW | Rockshelter | Chimbu | 3 | Bulmer 1966 ^†^ |
| NFA | NFA | Open | Eastern Highlands | 5 | Watson & Cole 1977 |
| NFB | NFB | Open | Eastern Highlands | 304 | Watson & Cole 1977 |
| NFC | NFC | Open | Eastern Highlands | 15 | Watson & Cole 1977 |
| NFD | NFD | Open | Eastern Highlands | 2 | Watson & Cole 1977 |
| Urufenafa | NIE | Open | Eastern Highlands | 4 | Swadling 1973^‡^ |
| Pundibasa | NMH | Open | Eastern Highlands | 10 | Swadling 1973^‡^ |

* revised count based on current 2014 analysis

^†^ Bulmer, S. (1966). *The Prehistory of the Australian New Guinea Highlands: A Discussion of Archaeological Field Survey and Excavations 1959-1960*. Unpublished MA thesis, The University of Auckland.

^‡^ Swadling, P. (1973). *The human settlement of the Arona Valley, Eastern Highland District, Papua New Guinea.* Papua New Guinea Electricity Commission, Boroko.
